# Corporatism as usual? – Staat und organisierte Wirtschaftsinteressen in der Coronakrise

**DOI:** 10.1007/s41358-021-00296-x

**Published:** 2021-11-09

**Authors:** Sebastian Fuchs, Detlef Sack

**Affiliations:** 1 Fakultät für Soziologie, Universität Bielefeld, Bielefeld, Deutschland; 2 Fakultät für Human- und Sozialwissenschaften, Institut für Politikwissenschaft, Bergische Universität Wuppertal, Wuppertal, Deutschland

**Keywords:** Covid 19-Krise, Organisierte Interessen, Krisenkorporatismus, Wirtschaftsverbände, Lobbying, Covid-19 crisis, Interest groups, Crisis corporatism, Business associations, Lobbying

## Abstract

Der Artikel widmet sich den Beziehungen zwischen Staat und organisierten Interessen der deutschen Wirtschaft in der Coronakrise 2020. Es werden zwei Fragen fokussiert, nämlich erstens, ob und wie Arbeitgeberverbände, Wirtschaftsverbände und -kammern sich in der Krise politisch artikulieren. Und zweitens, ob die Interaktion zwischen Regierung und organisierten Wirtschaftsinteressen den Charakter eines spezifischen ‚Krisenkorporatismus‘ annimmt. Ausgangspunkt sind Überlegungen zur pfadabhängigen Revitalisierung etablierter Interaktionsmuster in der Krise. Die Studie nimmt den Zeitraum zwischen März 2020 und Februar 2021 in den Blick. Auf Grundlage der Auswertung von Formen und Frequenz der Staat-Verbände-Interaktion werden drei Phasen identifiziert, denen sodann eingehende Fallstudien gewidmet werden. Für die Frühphase der Coronakrise (März–Juni 2020) werden a) die Angebote und politischen Forderungen von Arbeitgeberverbänden, Wirtschaftsverbänden und -kammern quantitativ ausgewertet und b) die inhaltliche Kongruenz zwischen Verbandsforderungen und den politischen Maßnahmen des Konjunkturpakets untersucht. Sodann wird die Ausformung des branchenspezifischen ‚Automobilkorporatismus‘ in der zweiten Phase (Juli–September 2020) in den Blick genommen. Schließlich wird für die dritte Phase (Oktober 2020–Februar 2021) eine Veränderung der Staat-Verbände-Interaktion anhand der Diskussionen um erneute Lockdowns und weitere wirtschaftspolitische Maßnahmen nachgezeichnet. Im Ergebnis identifiziert die Studie einen ‚Korporatismus ohne Verbrennungsmotor‘, der durch wechselseitige Ressourcenabhängigkeit, Vernetzungen und einen spezifischen ‚krisenkorporatistischen‘ Tausch zwischen organisierten Wirtschaftsinteressen und Staat geprägt und erhalten wird.

**Zusatzmaterial online:**

Zusätzliche Informationen sind in der Online-Version dieses Artikels (10.1007/s41358-021-00296-x) enthalten.

## Abstract (en)

The article explores the relationship between the state and organised business interests in Germany during the Covid-19 crisis of 2020. Two questions are addressed: Whether and how do employers’ associations, business interest associations and economic chambers articulate themselves in the context of the Covid-19 crisis? Does their interaction with the state follow the pattern of a dedicated ‘crisis corporatism’? The starting point is the concept of path-dependence and the revitalisation of established patterns of state-business-interaction in the crisis. The study focuses on the period between March 2020 and February 2021. Based on an investigation of the forms and frequencies of state-business-interactions, three phases are identified and then analysed in detailed case studies. For the early phase of the Covid-19 pandemic (March–June 2020) a) information offers and political demands of employers’ associations, business interest associations and economic chambers are examined quantitatively and b) the congruence between the groups’ demands and the political measures introduced with the economic stimulus package is investigated. For the second phase (July–September 2020) a case study of the branch-specific ‘automotive corporatism’ during the crisis is presented. Finally, the analysis focuses on the growing distance between business’ demands and government in the third phase (October 2020–February 2021). As a result, the article observes a ‘corporatism without combustion engine’, which is characterised and maintained by resource dependencies, network ties and a crisis-corporatist exchange between government and organised business interests.

## Einleitung

Die Studie untersucht die Beziehungen zwischen Staat und organisierten Interessen der deutschen Wirtschaft in der Coronakrise 2020/21. Dabei sind zwei Fragen von Belang, nämlich erstens, ob und wie Arbeitgeberverbände, Wirtschaftsverbände und -kammern (fortan auch: ‚Wirtschaftsverbände‘) sich in der Krise politisch artikulieren. Und zweitens, ob die Interaktion zwischen Regierung und Wirtschaftsverbänden den Charakter eines „Krisenkorporatismus“ (Rehder 2021) annimmt. Diese Überlegung geht grundsätzlich davon aus, dass auf den Schock der Ereignisse pfadabhängig im Rahmen etablierter Interaktionsmuster reagiert wird (Pierson 2004). Mit unserer Studie tragen wir konkret zum Forschungsstand bzgl. der Arbeitgeberverbände, Wirtschaftsverbände und Wirtschaftskammern in Deutschland und deren Leistungsfähigkeit bei (Schroeder und Weßels 2017). Wir schließen an Überlegungen zum Krisenkorporatismus (Urban 2012; Rehder 2021) an, über die wir empirisch wie auch theoretisch hinausgehen.

Der Artikel nimmt den Zeitraum zwischen März 2020 und Februar 2021 in den Blick. Wir gehen wie folgt vor: Die Diskussion des Forschungsstandes mündet in die Formulierung der Untersuchungshypothesen. Es folgt die Darstellung und Erläuterung der Interaktionen zwischen Staat und Wirtschaft auf Grundlage der Chronologie dokumentierter Treffen organisierter Wirtschaftsinteressen und der Bundesregierung. Hier können drei Phasen unterschieden werden, denen sodann eingehende Fallstudien gewidmet werden:

Zwischen *März und Juni 2020* (Verabschiedung des Konjunkturpakets) gab es eine intensive wirtschaftspolitische Aktivität, die auch durch die Interessenartikulation der Wirtschaftsverbände und -kammern geprägt wurde. Hier gehen wir auf die Informationsangebote und Forderungskataloge von 136 Verbänden und Kammern in der Frühphase der Coronapandemie ein. Als Schlüsselereignis und zugleich Abschluss dieser ‚Schockphase‘ der Pandemie untersuchen wir die Kongruenz verbandlicher Positionen und der politischen Maßnahmen des Konjunkturpakets.

Die relative Beruhigung der Coronalage mündete seit *Juli 2020* in eine Phase, in der im Rahmen bestehender korporatistischer Formate über strukturelle Probleme und staatliche Fördermaßnahmen beraten wurde. Für diese Phase nehmen wir insbesondere die Automobilindustrie und die Ausformung des branchenspezifischen „Automobilkorporatismus“ (Rehder und van Elten 2019; Rehder 2021) in der Coronakrise in den Blick.

Diese Phase überlappt sich teilweise mit der Krisenverschärfung ab Oktober 2020, den unterschiedlichen Unternehmenshilfen und der zunehmenden Kritik an diesen. Wir zeichnen hier zunächst nach, wie sich das Staat-Verbände-Verhältnis *im Oktober, November und Dezember 2020* deutlich verändert. Sodann gehen wir auf den Wirtschaftsgipfel vom 16.02.2021 als weiteres Schlüsselereignis ein, mit dem ‚die‘ Wirtschaftsverbände die Corona-Krisenpolitik der Bundesregierung (zwischenzeitlich) bilanzierten. Wir untersuchen für diese Phase insbesondere die Veränderung der Interaktion zwischen Verbänden und Politik.

Im Ergebnis identifizieren wir einen ‚Korporatismus ohne Verbrennungsmotor‘ (Sack et al. 2021), der durch wechselseitige Ressourcenabhängigkeit und Vernetzungen geprägt und erhalten wird.

Die Darstellung der Chronologie basiert auf der Auswertung einschlägiger Bundestagsdrucksachen und von Medienberichten. Den Erkenntnissen zu den Informationsangeboten und Forderungskatalogen der deutschen Arbeitgeberverbände, Wirtschaftsverbände und -kammern liegt eine quantitative Analyse der entsprechenden Websites zugrunde (Fuchs et al. 2021). Die Untersuchung der Verbandspositionen und Interaktionsmuster in den drei Phasen basiert auf der qualitativen Analyse der manifesten Inhalte (Miles et al. 2014) von Verbandsstellungnahmen (*n* = 98), validiert durch die Auswertung von Medienberichterstattung.^1^

## Organisierte Interessen der deutschen Wirtschaft – Forschungsstand

Ein erster Strang der Forschung zu organisierten Wirtschaftsinteressen in Deutschland hat sich, neben Übersichtswerken (Sebaldt und Straßner 2004; Schroeder und Weßels 2017), in den vergangenen Jahren auf organisationsbezogene Fragestellungen fokussiert. Im Mittelpunkt stehen ihre Organisierbarkeit (Behrens (2011) für Arbeitgeberverbände), ihr Organisationserhalt und Wandel im Kontext sich verändernder Umwelten (Sack (2021) für Industrie- und Handelskammern; Grote et al. (2008) und Kohler-Koch et al. (2021) für Industrieverbände), ihre Konkurrenzbeziehungen (Vorholt 2019) oder die Veränderung der Zahl von Verbänden im Zeitverlauf (Klüver und Zeidler 2019). In weiteren Arbeiten geht es um interne Organisationsdynamiken und die Legitimität aus Sicht der Mitgliedsunternehmen (Sack et al. 2014 und van Elten 2018 für Wirtschaftskammern). Die organisierten Interessen der deutschen Wirtschaft werden auch entlang ihrer spezifischen Funktionen differenziert. Für die Arbeitgeberverbände geht es dabei wesentlich um die Tarifpolitik (Silvia und Schroeder 2007; Behrens 2017; Haipeter 2016, 2017; Silvia 2017). Hierbei steht die in der deutschen Wirtschaftsverbandsforschung etablierte Unterscheidung dreier Typen organisierter Wirtschaftsinteressen – Arbeitgeberverbände, Wirtschaftsverbände und Wirtschaftskammern (Grote et al. 2007; Schroeder und Weßels 2017; Kohler-Koch et al. 2021) – jedoch zunehmend in Frage. So erodierte die Distinktion von Arbeitgeberverbands- und Wirtschaftsverbandsfunktionen durch einen generellen Rückgang der Tarifbindung, staatliche Interventionen in die Lohnpolitik aber auch produktmarktbezogene Regulierungen (Ellguth und Kohaut 2020; Lesch 2017). Die Wirtschaftskammern unterlagen in den letzten 15 Jahren einem institutionellen Wandel, der mit der verwaltungsrechtlichen Einhegung ihrer Interessenartikulation einherging (Sack 2021; Kluth 2021). Diese Arbeiten sind für unsere Untersuchung insofern von Interesse, als dass sie Stabilität und Wandel der wirtschaftsverbandlichen Organisation in den Blick nehmen.

Ein zweiter Forschungsstrang befasst sich mit Public Policy, Politikwandel und organisierten Interessen. Im Kern geht es (Stichwort: Inside- und Outside-Lobbying) um ‚alte‘ Fragen der Politikwissenschaft: Welche Verbände werden in den Policy-Prozess einbezogen? Welchen Einfluss üben organisierte Wirtschaftsinteressen auf politische Entscheidungen aus? Dabei werden sowohl die Verhandlung zwischen organisierten Wirtschaftsinteressen und Staat in Politikfeldern untersucht (Rehder et al. 2009; Schiffers 2019) als auch ihre langfristige Rolle für die Gestaltung von Politik adressiert (Paster 2015). Sodann geht es um den Einfluss organisierter Wirtschaftsinteressen auf Regulierung, den sie gegenüber unterschiedlichen Adressaten des politischen Systems versuchen geltend zu machen (z. B. Sack und Fuchs 2014; Dür et al. 2019) bzw. um die Kongruenz ihrer Präferenzen mit (Teilen) der Bevölkerung (Pakull et al. 2020). Für deutsche Wirtschaftsverbände kommen Eising und Spohr (2017) und Cross et al. (2021) zu dem Schluss, dass diese in Anhörungen häufiger als andere organisierte Interessen erfolgreich auf Änderungen von Gesetzesentwürfen im deutschen Bundestag hinwirken. Döhler (2020) weist auf die Bedeutung des Verhältnisses organisierter Interessen zur Ministerialverwaltung hin. Er identifiziert eine „reziproke[.] Beziehung“ (Döhler 2020, S. 22), die sich deutlich von den Vorstellungen einseitiger Einflussnahme im Sinne des Lobbying-Diskurses unterscheidet. An diesen Diskussionsstrang knüpfen wir mit der Überprüfung an, ob sich Einfluss organisierter Wirtschaftsinteressen anhand einiger Schlüsselsituationen in der Coronakrise plausibilisieren lässt. Dabei streben wir keine ‚Messung‘ des Einflusses (Dür et al. 2019; Sack 2017) oder des ‚Zugangs‘ zu Medien und politischen Institutionen (für die Coronakrise: Junk et al. 2021)^2^ an, sondern überprüfen die Kongruenz und Parallelität der Positionen organisierter Wirtschaftsinteressen und wirtschaftspolitischer Programme der Bundesregierung.

Es geht bei der Einbeziehung der organisierten Interessen der Wirtschaft jedoch nicht nur um die Einflusslogik (Schmitter und Streeck 1999) und das Lobbying (Baumgartner et al. 2009; Klüver 2012; Dür et al. 2019), sondern auch um die „participation in public policy“ (Traxler 2010, S. 163), also die Integration von Verbänden in öffentliche Politik und damit um ihre Leistungs- und Problemlösungsfähigkeit. Diese Überlegungen schließen an die Neo-Korporatismusforschung an, die auf die Ausgestaltung von Politikverhandlung und der Strukturen der Interessenvertretung hingewiesen hat. Systematisch sind zwei Analyseebenen zu unterscheiden (Schmitter 1982, S. 262): So geht es erstens um die Bedingungen korporatistischer Politikverhandlung, d. h. das Vorhandensein großer und repräsentationsstarker Verbände. Zweitens geht es um die Ausgestaltung der Interessenintermediation und -konzertierung, d. h. die regelmäßige und bevorzugte Einbeziehung dieser (wenigen) Verbände in etablierten (meist nicht öffentlichen) Foren und Formaten (vgl. von Winter 2014, S. 182). Diese (bevorzugte) Einbeziehung wird seitens der Verbände – im Sinne eines korporatistischen ‚Tauschs‘ (Lehmbruch 1978, 1983) – durch ihre Beteiligung an und die Unterstützung der Politikimplementation ‚entgolten‘. Während in den vergangenen Jahren mehrfach die ‚Erosion‘ korporatistischer Strukturen – u. a. durch eine ‚Pluralisierung‘ der Akteursgruppen – diskutiert wurde (von Winter und Willems 2007; von Winter 2014; Haipeter 2016), legen die Erkenntnisse zur Finanz- und Wirtschaftskrise 2008/09 einerseits und zu den Strukturen deutscher Wirtschaftsverbände andererseits (Kohler-Koch et al. 2021) nahe, dass korporatistische Strukturen und Praktiken nach wie vor hoch relevant sind.

Zusammenfassend ist Korporatismus nicht einfach nur als Verhandlungskoordination und „administrative Interessenvermittlung“ (Lehmbruch 1987) zwischen Regierung und (wenigen) Verbänden definiert, sondern bedeutet auch, dass diese problemlösungs- und implementationsfähig sind. Damit wird die *krisenspezifische* Problemlösungsfähigkeit des Korporatismus analytisch interessant, über die bislang wenig bekannt ist. Für die Finanz- und Wirtschaftskrise 2008/09 identifizierte Urban (2012) einen distinkten „Krisen-Korporatismus“ (S. 229). Er fokussierte auf die ‚klassische‘ Kooperation zwischen Staat, Gewerkschaften und organisierten Wirtschaftsinteressen und zeigte, dass die Konzertierung grundsätzlich zur Krisenbewältigung beitrug. Zu einem ähnlichen Ergebnis, nämlich der hohen Bedeutung neokorporatistischer Interessenkonzertierung in 2008/09, kamen (in international vergleichender Perspektive) auch Eichhorst und Weishaupt (2013). Für den Fokus dieser Untersuchung, die Coronakrise (2020/21), diagnostiziert Rehder (2021) mit Blick auf organisierte Wirtschaftsinteressen eine pfadabhängige Praxis „krisenkorporatistischer“ Konzertierung und Steuerung, die an die Krisenerfahrungen von 2008/09 – z. B. mit Blick auf das Kurzarbeitergeld – anschließt (Rehder 2021, S. 217). Die Einbindung von Verbänden eröffne dem Staat ein wesentliches „Politikinstrumentarium“ (Rehder 2021, S. 217) für die Bewältigung (ökonomischer) Krisen. Mit Blick auf die Interessenintermediaton zeige sich, dass „unter wirtschaftlichen Krisenbedingungen die korporatistische Tradition [..] immer wieder revitalisiert wird“ (Rehder 2021, S. 217).

Wir knüpfen hier an diesen Begriff des ‚Krisenkorporatismus‘ im Sinne Rehders an. Unser Interesse richtet sich damit auf einen Ausschnitt korporatistischer Interessenintermediation, nämlich die Interaktion organisierter Wirtschaftsinteressen auf der einen und der deutschen Bundesregierung auf der anderen Seite in einer zeitlich begrenzten Krisensituation (Coronakrise 2020/21).^3^ Wir stehen weder an, Korporatismus in seiner ganzen Bandbreite zu vermessen, noch können wir hier einen zeitlichen Vergleich leisten. Der Beitrag fokussiert auf eine krisenspezifische Ausprägung des Korporatismus, der sich von der regelmäßigen korporatistischen Konzertierung unterscheiden kann; dessen genaue Ausformung jedoch empirisch zu bestimmen ist. Gemeinsam mit der policy-analytischen Frage, ob und wie organisierte Wirtschaftsinteressen auf die Krisenpolitik einwirken, werden also die Muster der Einbeziehung organisierter Wirtschaftsinteressen in der spezifischen Situation der Coronakrise untersucht.

Auf Grundlage der vorangegangenen theoretischen Überlegungen können zwei untersuchungsleitende Hypothesen abgeleitet werden. Die erste Hypothese greift die Überlegungen zum Krisenkorporatismus (Rehder 2021) auf und pointiert diese wie folgt:

Aufgrund des Zeit- und des Problemdrucks wird in der Krise korporatistische Handlungskoordination konfliktvermeidend und effizienzorientiert pfadabhängig „revitalisiert“. (H1)

Im Anschluss an diese Überlegungen, aber auch die Hinweise zur erwarteten Problemlösungsfähigkeit von Verbänden bei korporatistischer Handlungskoordination lässt sich eine zweite Hypothese formulieren:

Aufgrund der gesellschaftlichen Problemlösungsfähigkeit ressourcenstarker Verbände werden diese von der Regierung aktiviert und in die Definition und Umsetzung der Krisenstrategie einbezogen. (H2)

Vor dem Hintergrund ihrer (einstmals?) starken Stellung gilt ein besonderer Fokus in der Debatte um den Krisenkorporatismus der Automobilindustrie. Rehder (2021) diagnostiziert hier längerfristige mit der Dieselgate-Affäre verbundene „Legitimitätsverluste“ und eine Erosion des „Automobilkorporatismus“ (S. 217). Hieran anschließend interessiert uns, ob während der Krise tatsächlich eine ‚geschwächte‘ Rolle der Automobilindustrie zu identifizieren war. Dies ist eine Überprüfung für die konkrete Situation. Eine grundlegende Analyse der Veränderungen des „Automobilkorporatismus“ bedarf längerer Zeiträume. Gleichwohl ist die Beobachtung der Staat-Verbände-Beziehungen in diesem Sektor ein wichtiger Baustein, um Kontinuitäten, Anpassungen und Veränderungen zu erfassen.

Festzuhalten bleibt, dass es bislang an einer eingehenden empirischen Überprüfung der dargestellten Überlegungen zum aktuellen Krisenkorporatismus fehlt. Hier liegt dann auch der Mehrwert der Studie.

## Drei Phasen der Staat-Verbände-Interaktion

Um die Frage nach dem „Krisenkorporatismus“ bzw. dessen Plausibilität in den Blick zu nehmen, haben wir zunächst Frequenz und Formen der nachvollziehbaren Interaktion zwischen Staat und (assoziierter) Wirtschaft (vgl. Rehder 2021, S. 216–217) zwischen März 2020 und Februar 2021^4^ ausgewertet. Wir fokussieren auf die Interaktion von *Arbeitgeberverbänden, Wirtschaftsverbänden und Wirtschaftskammern (Handwerkskammern, Industrie- und Handelskammern)* mit der *Bundesregierung*. Auf die Interaktion mit dem Parlament oder auf die EU- und Bundesländerebene konnten wir in den folgenden Untersuchungsschritten ebenso wenig eingehen wie auf die Rolle der Gewerkschaften in der Krise (s. oben). Die Erhebung basiert auf der Recherche auf den Websites der Bundesministerien und der Auswertung einschlägiger Bundestagsdrucksachen (Onlinematerial 1). Wir kommen zu zwei Ergebnissen: Zum einen lassen sich unterschiedliche Typen von Kooperationsformaten identifizieren. Zum anderen zeigt sich eine Periodisierung der Staat-Verbände-Interaktion im ersten Jahr der Coronapandemie.

Mit Blick auf die Interaktionsformate müssen *a) solche Treffen und Foren, die der (ad-hoc) Bearbeitung akuter Krisenfolgen dienen*, von *b) etablierten (korporatistischen) Formaten der Interessenkonzertierung unterschieden werden*, die bereits vor der Krise eingerichtet wurden (z. B. Allianz für Aus- und Weiterbildung). Letztgenannte sind inhaltlich vor allem auf Fragen der Branchenentwicklung (z. B. Digitalisierung, Verkehrswende) in mittel- bis langfristiger Perspektive ausgerichtet. Im Kontext der Coronakrise werden damit jedoch *selbstverständlich* immer auch Maßnahmen zur Krisenfolgenbewältigung thematisiert. Es geht uns hier also um den *Fokus* der Gespräche und nicht um eine exklusive Zuordnung.

Die Periodisierung der Staat-Verbände-Interaktion ist mit der wirtschaftspolitischen Aktivität der Bundesregierung kongruent (Sack et al. 2021). Diese orientiert sich wesentlich an der Entwicklung des Infektionsgeschehens, da die restriktiven, die ökonomische Aktivität beschränkenden Maßnahmen der Bundesregierung sich am Hygieneschutz ausrichteten. Die *erste Phase (März–Juni 2020)* ist von der engen und regelmäßigen Abstimmung der Wirtschaftsverbände mit der Bundesregierung geprägt. Inhaltlich war die Interaktion von der unmittelbaren Bewältigung der Coronakrise bestimmt. Es dominierten Treffen, die eigens zur Bewältigung krisenspezifischer Fragen der Wirtschaftspolitik eingerichtet wurden. Hierzu zählen insbesondere die ‚Verbänderunden‘ im Bundeskanzleramt. Die Kooperation folgte einem klaren Muster: Einbezogen wurden Verbände, die einem ‚korporatistischen Ideal‘ der Interessenvertretung entsprechen, also große Branchen- und Spitzenverbände (z. B. BDA, BDI, DIHK, HDE)^5^ mit einer hohen Repräsentationsstärke und ‚Verpflichtungsfähigkeit‘, die zugleich für die Krisenbekämpfung relevante Wirtschaftsbereiche repräsentieren. Formate staatlich-verbandlicher Koordinierung, die bereits vor dem Beginn der Coronakrise eingerichtet wurden, wurden nur am Rande zur Krisenbewältigung reaktiviert. Dies gilt in dieser ersten Phase v. a. für die mittelstandspolitische Interessenkonzertierung; so wurden die entsprechenden Verbände über das ‚Netzwerk Mittelstand‘ des Bundeswirtschaftsministeriums (BMWE) zweimal nachweislich eingebunden.^6^

Die etablierten Formate wurden aber dann vor allem mit Beginn der *zweiten Phase (Juli–Oktober 2020)* wichtiger. Nicht zuletzt aufgrund des deutlichen Rückgangs der Infektionszahlen dominierten sie über die Sommermonate und im Frühherbst, während die Zahl solcher Gespräche, Treffen und ‚Gipfel‘ deutlich nachließ, die sich mit akuter Krisenbewältigung befassten. Nur der *Handelsverband Deutschland (HDE)* kam mit dem BMWE zu einem dezidiert Corona-bezogenen ‚Austausch‘ zusammen. Im Oktober fand (nach langer Pause) ein weiterer ‚Verbändegipfel‘ in größerer Runde statt. Zu den dann quantitativ überwiegenden erprobten korporatistischen Formaten zählen insbesondere die von hoher medialer Aufmerksamkeit begleiteten Treffen der Bundesregierung mit der Automobilindustrie im Rahmen der etablierten *‚Konzertierten Aktion Mobilität‘* (Deutscher Bundestag, BT-Drs. 19/11025). Dies gilt auch für den *‚Runden Tisch Luftfahrtindustrie‘* und den *‚Nutzfahrzeuggipfel‘*, die zwar auch im Zeichen der Coronapandemie standen, jedoch insbesondere Fragen der branchenspezifischen Entwicklung thematisierten.

Die *dritte Phase der Staat-Verbände-Interaktion (November 2020–Februar 2021)* begann mit dem abermaligen Lockdown im Herbst 2020 infolge der erneuten Infektionswelle. Diese Phase ist durch eine auffällige Inaktivität^7^ gekennzeichnet: Weder fanden hier nennenswerte ad-hoc-Krisentreffen statt, noch wurde eine große Zahl etablierter Formate einberufen. Lediglich zwei ‚Verbändegespräche‘ im Oktober 2020 und Februar 2021 sowie obligatorische Antrittsbesuche neuer Verbandspräsidenten im BMWE (Wolf, Gesamtmetall und Dulger, BDA) sind hier zu verzeichnen. Kennzeichnend für diese Phase sind die relative Begrenzung der Restriktionen auf die ‚kundennahen‘ Bereiche des Handels, der Gastronomie und des Tourismus sowie die Einführung und Fortsetzung distributiver Maßnahmen der Bundesregierung („Überbrückungshilfen“).

**Figure 1:**
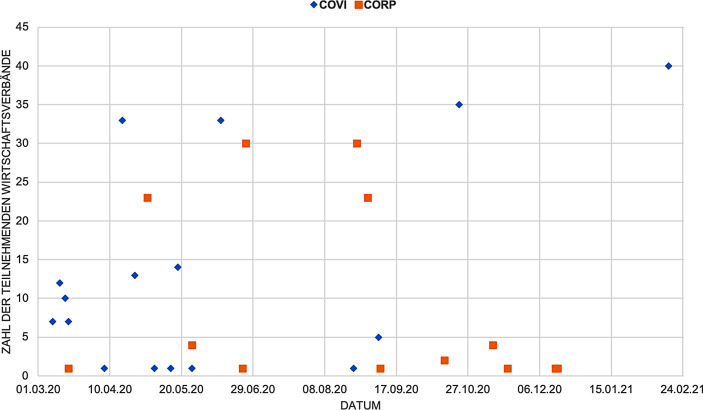

Zusammenfassend lässt sich erstens mit Blick auf die Formen und Teilnehmer der Staat-Verbände-Interaktion in allen drei Phasen ein ausgeprägter Krisenkorporatismus identifizieren. Mit Blick auf die Einbeziehung repräsentations- und ressourcenstarker Verbände knüpft er an etablierte korporatistische Praktiken an, ist jedoch auch spezifisch. So ist die *Zahl* der einbezogenen Verbände kontinuierlich höher als in ‚klassischen‘ korporatistischen Formaten. Überdies erfolgt die Interaktion öffentlich durchaus sicht- und nachvollziehbar. Beides kann als eine spezifische Reaktion auf die Coronakrise verstanden werden. Denn diese zeichnet sich, auch im Vergleich mit vorherigen Krisensituationen (z. B. Finanzkrise 2008/09), sowohl durch eine *umfassende* Betroffenheit und ein damit einhergehendes Interesse von Gesellschaft wie Wirtschaft als auch durch eine Vielzahl bereichs*spezifischer* Problemstellungen (z. B. Betriebsschließungen in Handel und Gastronomie vs. Beeinträchtigung von Lieferketten in der Industrie) aus. Schließlich zeigt sich zweitens, dass die Frequenz der Interaktion in den drei Phasen deutlich variiert (Abb. 1). Diese ‚Bestandsaufnahme‘ der Interaktionsmuster stellt die Hintergrundfolie für die nachfolgende Analyse der Staat-Verbände-Beziehungen in den drei Phasen dar.

## Staat-Verbände-Beziehungen in der Coronakrise

### Phase 1: Das Konjunkturpaket

In der Frühphase der Coronakrise unterschieden sich 136 deutsche Arbeitgeberverbände, Wirtschaftsverbände und Kammern^8^ anhand der Online-Angebote und politischen Statements deutlich in ihrem Aktivitätsniveau: Während sich 38 Verbände und Kammern ausgesprochen passiv verhielten, d. h. höchstens eine Information vorgehalten bzw. politische Forderung gestellt haben, waren 62 Verbände und Kammern mit mehr als fünf Informationen und/oder politischen Forderungen präsent. Dabei fällt auf, dass die Wirtschaftskammern vergleichsweise stark präsent waren, wenn es um Dienstleistungsangebote und Informationen für ihre Mitglieder und die Öffentlichkeit ging. Dies liegt an ihrer institutionellen Funktion und relativen Ressourcenstärke (Sack 2021). Ebenfalls erwartungsgemäß orientierten sich die politischen Forderungen mehrheitlich an der Frage nach finanziellen Unterstützungen für Unternehmen. Im Bereich der Informationen waren quantitativ branchenspezifische Informationen über die Virusprobleme, arbeitsrechtliche/-organisatorische Belange und die Betriebsorganisation bestimmende Themen. Die Forderungen nach der finanziellen Unterstützung von Unternehmen stechen quantitativ besonders hervor. Nachrangig sind in dieser ersten Phase der Pandemie Forderungen nach Änderungen bei den Arbeitsbeziehungen (Kurzarbeit, Sozialversicherungsbeiträge oder Mindestlohn) und nach der Aufhebung des Lockdowns. Forderungen nach steuerrechtlichen Erleichterungen und der raschen Umsetzung der staatlichen Finanzhilfen für Unternehmen wurden in diesem Zeitraum von mehr als 35 Wirtschaftsverbänden und -kammern artikuliert. Mit Blick auf die Maßnahmen der deutschen Bundesregierung gibt es hier eine hohe Kongruenz bei den kurzfristig angelegten Policy-Maßnahmen. Die faktische Aussetzung des Insolvenzrechts und die staatliche Beteiligung an Unternehmen über den Wirtschaftsstabilisierungsfonds spielten keine besondere Rolle. Es deutete sich aber bereits an, dass die konkrete Umsetzung der Policies von den Verbänden thematisiert wird (Fuchs et al. 2021).

Für die ‚Schockphase‘ ab März 2020 funktioniert der Krisenkorporatismus also in großen Teilen in Form eines unmittelbaren Informations- und Serviceangebots für die Mitglieder und von Forderungen, um die aktuelle Überlebensfähigkeit der Unternehmen (und damit Arbeitsplätze) zu sichern. Diese korrespondieren mit der Bereitschaft der Regierung, distributive Policies mit einem hohen Finanzmitteleinsatz als Krisenstrategie zu verfolgen. Es ist insoweit eine wechselseitige Ressourcenabhängigkeit sichtbar, als die Wirtschaftsverbände und -kammern seitens der Politik für die Bereitstellung von Informationen und Dienstleistungen genutzt werden, die insgesamt der Implementation der Infektionsschutzmaßnahmen^9^ dienen. Wissen und (implementationsrelevante) Leistungen der Verbände können in dieser (informierten) Weise regierungsseitig nicht unmittelbar zur Verfügung gestellt werden. Sie werden mit distributiven, krisenentschärfenden Maßnahmen für Unternehmen entgolten. Es handelt sich insofern um einen ‚krisenkorporatistischen‘ Tausch (vgl. Lehmbruch 1978, 1983): (a) Öffentliche Unterstützung und damit Legitimierung der Regierungspolitik, (b) Informationsangebote für Unternehmen, (c) Implementationsunterstützung und (d) Arbeitsplatzsicherung auf Seiten der Wirtschaft und ihrer Verbände werden gegen finanzielle Unterstützung und die Bereitschaft, auf weitreichende Forderungen der Verbände einzugehen, seitens der Regierung getauscht.

Dies zeigt sich auch daran, dass die Policies der Bundesregierung und die Forderungen der Wirtschaftsverbände inhaltlich stark kongruent sind. Ein besonders eindrückliches Beispiel ist das Konjunkturpaket als umfassendste wirtschaftspolitische Maßnahme im Frühjahr 2020 (Koalitionsausschuss 2020). Die Spitzenverbände der Wirtschaft – nachfolgend werden der *Bundesverband der Deutschen Industrie* (BDI), der *Deutsche Industrie- und Handelskammertag* (DIHK) sowie die *AG Mittelstand* (AGM) einbezogen^10^ – veröffentlichten im Vorlauf zahlreiche Vorschläge für ökonomische Stimuli (BDI, 28.05.2020; DIHK, 02.06.2020; AGM, 02.06.2020b).^11^ Hierzu zählten übereinstimmend und insbesondere steuerliche Anreize wie eine Ausweitung des steuerlichen Verlustrücktrags oder verbesserte Abschreibungsmöglichkeiten, um den „Spielraum für neue private Investitionen [zu] erweitern“ (DIHK, 02.06.2020). Ebenso wurden weitreichende öffentliche Investitionen als „ein Türöffner für die private Investitionstätigkeit“ (BDI, 28.05.2020, S. 4), vor allem im Bereich von E‑Mobilität und Digitalisierung gefordert und eine beschleunigte Verwaltungsdigitalisierung einhellig angemahnt. BDI und AGM forderten zudem eine Deckelung der Sozialversicherungsbeiträge, die AGM setzte sich für eine finanzielle Unterstützung von Ausbildungsbetrieben und der Kommunen ein. All diese Maßnahmen finden sich im Konjunkturpaket der Bundesregierung (s. Tab. 1).^12^

**Table 1:** 

Maßnahme	Geschätzte fiskalische Kosten	DIHK	BDI	AG Mittelstand
*Steuerliche Maßnahmen, Liquiditätshilfen und Finanzierungshilfen*				
Temporäre Absenkung des Mehrwertsteuersatzes von 19 % auf 16 % (bzw. 7 % auf 5 % beim ermäßigten Satz)	20 Mrd. €	o	o	o
Senkung der EEG-Umlage ab 2021 über Zuschüsse aus dem Bundeshaushalt	11 Mrd. €	+++	−	++
Deckelung der Sozialversicherungsbeiträge durch steuerfinanzierte Zuschüsse auf maximal 40 %	5,3 Mrd. €	o	+++	+++
Erweiterung des steuerlichen Verlustrücktrags für 2020 und 2021 auf max. 5 Mio. € (bzw. 10 Mio € bei Zusammenveranlagung)	2 Mrd. € (Verschiebungseffekt)	++	++	++
Verbesserte Abschreibungsmöglichkeiten und Forschungszulagen für 2020 und 2021	7 Mrd. €	++	+++	++
Kinderbonus in Höhe von 300 € pro Kind und Verdoppelung der Freibeträge für Alleinerziehende	5 Mrd. €	o	o	o
*Förderprogramme und Subventionen*				
Bonusprogramm für Investitionen in Innovationen und neue Antriebstechnologie in der Autoindustrie	2 Mrd. €	o	+++	o
Erhöhung der Kaufprämie für Elektroautos	2,2 Mrd. €	o	++	o
Unterstützung der Flottenerneuerung bei Bussen, Lastkraftwagen, Flugzeugen und Schiffen	3,2 Mrd. €	o	+++	o
Azubi-Prämienprogramm	0,5 Mrd. €	o	o	+++
*Öffentliche Investitionen in Infrastruktur und Zukunftstechnologien, Stützung der kommunalen Finanzen*				
Vorgezogene öffentliche Investitionen in die Digitalisierung der öffentlichen Verwaltung und Sicherheits- und Rüstungsprojekte	10 Mrd. €	++	++	++
Investitionen in Zukunftstechnologien (Wasserstofftechnik, Quantentechnologie, künstliche Intelligenz und Kommunikation)	13 Mrd. €	+	++	+
Investitionen in Elektromobilität	2 Mrd. €	+	++	+
Modernisierung der Bahn durch Kapitalerhöhung des Bundes	5 Mrd. €	+	+	+
Beschleunigung des Ausbaus eines flächendeckenden 5G Netzes	5 Mrd. €	+	+	++
Energetische Gebäudesanierung	2 Mrd. €	+	++	+
Ausbau von Kitas und Ganztagsschulen	3 Mrd. €	+	+	+
Stützung der Kommunen	12,5 Mrd. €	o	o	++

*Code-Schlüssel:* − explizit gegenteilige Forderung o. keine Forderung, + allgemeine Forderung einer Maßnahme im jeweiligen Bereich (z. B. ‚Investitionen in Infrastruktur‘), ++ konkrete Forderung wird zum Teil erfüllt, +++ Forderung weit überwiegend erfüllt oder Maßnahmen und Forderung identisch *(im Bereich „Öffentliche Investitionen“ wurde auch dann „++“ codiert, wenn die Forderung nur inhaltlich ohne Nennung konkreter Maßnahmen artikuliert wurde [z.* *B. Verwaltungsdigitalisierung])*

*Quellen Verbändepositionen:* DIHK, 02.06.2020, „Wirtschaftsstandort bewahren – Zukunftschancen ergreifen“; BDI, 28.05.2020, „Eckpunkte für ein modernes und effektives Konjunkturpaket“; AG Mittelstand, 02.06.2020, „Wertschöpfung und Beschäftigung mit dem Mittelstand stärken!“

*Quellen Maßnahmenübersicht:* Dorn et al. (2020); Sachverständigenrat (2020)

Abweichungen zeigen sich vor allem mit Blick auf die Nachfrageseite. So war z. B. die Auszahlung eines ‚Kinderbonus‘ keine dezidierte Forderung der Spitzenverbände. Dennoch stimmen auch diese Maßnahmen in ihrer Zielsetzung mit Verbandsforderungen, z. B. nach einer vorrübergehenden Senkung der Körperschafts- und Einkommensteuern (BDI) oder der *vollständigen* Abschaffung des Solidaritätszuschlags (BDI, AGM), überein.^13^

Auch die Bewertung der Beschlüsse fiel weit überwiegend positiv aus. So war die ‚Anerkennung‘ politischer Entschlossenheit und ‚Tatkraft‘ kennzeichnend, auch wenn Detailfragen kritisiert wurden (z. B. DEHOGA, 04.06.2020). Dies gilt auch für die Mittelstandsverbände, die das Fehlen zukunftsorientierter struktureller Maßnahmen und die langfristigen Kosten kritisierten (FamUnt, 04.06.2020; BVMW, 04.06.2020; ZGV, 04.06.2020).

Abseits der grundsätzlich zustimmenden Positionen waren es nur Verbände der Tourismuswirtschaft, die vor dem Hintergrund der hohen Betroffenheit der Branche umfassend Kritik übten (BTW u. a., 04.06.2020). Diese machte sich vor allem am Fehlen einer für die Branche essentiellen Regelung zu Kundengeldrückerstattungen und andauernden Betriebsverboten fest. In diesem Fall fehlte anscheinend eine entsprechende ‚Vergütung‘ für die Kooperation und Implementationshilfe (Umsetzung des Infektionsschutzes, Arbeitsplatzerhalt) der Verbände; der krisenkorporatistische Tausch funktioniert in diesen Fällen nicht.

### Phase 2: Der Fall der Automobilindustrie

Die zweite Phase der Staat-Verbände-Interaktion ist durch branchenspezifische Interessenkonzertierung und Handlungskoordination in etablierten korporatistischen Formaten geprägt. Wir fokussieren hier auf den Einzelfall der Automobilindustrie. Denn diese stand mit eigenen ‚Autogipfeln‘, sowohl in der Frühphase der Pandemie als auch im weiteren Verlauf des Jahres 2020, im besonderen Fokus. Vor dem Hintergrund der Diskussion über eine schleichende Erosion und Legitimitätsverluste des „Automobilkorporatismus“ (Rehder und van Elten 2019; Rehder 2021; s. oben), überprüfen wir, ob eine gesteigerte Distanz zwischen Automobilindustrie und Politik für die Coronakrise belegt werden kann.

Bereits bei der Diskussion um das Konjunkturpaket ab April 2020 stand die Automobilindustrie im Fokus. Der *Verband der Automobilindustrie (VDA)* trat öffentlich für eine weitreichende ‚Kaufprämie‘ für Automobile – analog zur Umweltprämie (‚Abwrackprämie‘) aus der Finanz- und Wirtschaftskrise 2008/09 – ein. Der Verband forderte explizit *auch* die finanzielle Förderung des Kaufs von Automobilen mit ‚modernem‘ Verbrennungsmotor (DLF 2020). Im Kontext der virulenten klimapolitischen Diskussionen stieß diese Forderung in der Öffentlichkeit jedoch auf deutlichen Widerspruch (SZ 2020a). Daran änderte auch die Fürsprache der Ministerpräsidenten der drei ‚Autoländer‘ Baden-Württemberg, Bayern und Niedersachsen (SZ 2020a) sowie des industriellen Spitzenverbands BDI^14^ (BDI, 28.05.2020, S. 5) wenig. Eine Kaufprämie auch für Verbrennungsmotoren wurde weder nach einem öffentlichkeitswirksamen ‚Autogipfel‘ (05.05.2020) noch mit dem Konjunkturpaket (03.06.2020) beschlossen. Dies wurde als Scheitern des VDA, als „Stromschlag für die Lobby“ (SZ 2020b), wahrgenommen. Der Verband wie Unternehmensvertreter zeigten sich „mit dem Ergebnis der Verhandlungen unzufrieden“ (FAZ.net 2020b; VDA, 04.06.2020). Allerdings handelt es sich, so das Ergebnis unserer Analyse, um eine dezidierte Strategie des VDA – die Kaufprämie *auch* für Verbrennungsmotoren war nicht mehr als ein ‚Lobbying-Dummy‘. Denn das Scheitern der Forderung ließ die Automobilindustrie in der Öffentlichkeit als Verliererin dastehen, während sie erhebliche Mittel aus dem Konjunkturpaket – ebenso wie im weiteren Verlauf des Jahres 2020 – für sich sichern konnte. Insbesondere der Eindruck, es sei zu einem ‚Bruch‘ zwischen VDA und Bundesregierung gekommen, trifft nicht zu. Denn der Verband wurde nach dem als gescheitert betrachteten Autogipfel eng in die Vorbereitung des Konjunkturpakets eingebunden; mit der IG Metall wurde er in eine „Arbeitsgruppe für konjunkturbelebende Maßnahmen“ des BMWE berufen (BMWE 2020; FAZ.net 2020a). Und mit dem Konjunkturpaket wurde nicht nur die (von den Automobilherstellern kofinanzierte) ‚Innovationsprämie‘ für E‑Autos beschlossen, sondern auch eine umfassende – direkte wie indirekte – finanzielle Förderung der Automobilindustrie im Umfang von 8,1 Mrd. € (Koalitionsausschuss 2020).^15^ Hierzu zählen verschiedene Programme zur Modernisierung von Nutzfahrzeugflotten, der Ausbau der Ladeinfrastruktur oder die Förderung von FuE-Projekten für Elektromobilität und Batteriezellforschung (s. Tab. 2).^16^

**Table 2:** 

Datum	Vorgang	Ergebnisse
07.04.2020	**Treffen im BMWE zum ‚Beitrag der Automobilindustrie zum Gesundheitsschutz‘**	Öffentliche **‚Würdigung‘** der Unterstützung der Automobilindustrie bei der Bewältigung der Coronakrise (Spenden, Bereitstellung von Personal und Schutzausrüstung etc.) (BMWE und VDA 2020)
05.05.2020	**Autogipfel (apl.) im Kanzleramt**	Einrichtung einer „Arbeitsgruppe für konjunkturbelebende Maßnahmen“ (BMWE 2020); keine weiteren Entscheidungen
14.05.2020 26.05.2020	**Arbeitsgruppe für konjunkturbelebende Maßnahmen**	Vorbereitung Maßnahmen Konjunkturpaket, *genaue Vorschläge nicht bekannt*
03.06.2020	**Beschluss Konjunkturpaket (Koalitionsausschuss)**	**Insgesamt 8,1** **Mrd.** **€** Förderung (direkt/indirekt), u. a. Innovationsprämie, Flottenaustauschprogramme, Forschungsförderung E‑Mobilität, Ladeinfrastruktur (Koalitionsausschuss 2020; Deutscher Bundestag, BT-Drs. 19/25739)
08.09.2020	**Autogipfel („Konzertierte Aktion Mobilität 3“)**	Einsetzung Arbeitsgruppen zur Umsetzung des Konjunkturpakets und v. Unterstützungsprogrammen für Mittelständler, beabsichtigte Gesetzgebung Bezahlsystem Ladestationen, Absichtserklärung Ladeinfrastruktur (Bundesregierung 2020a)
12.11.2020	**Nutzfahrzeuggipfel**	u. a. Kostenzuschuss Flottenmodernisierung, Schaffung von Ladeinfrastruktur, (technologieneutrale) Maut-Ermäßigung bei geringerem Schadstoffausstoß – *die Fördermaßnahmen werden auf 4* *Mrd.* *€ taxiert*^*a*^ (BMVI 2020b)
17.11.2020	**Autogipfel („Konzertierte Aktion Mobilität 4“)**	**Insgesamt 3,2** **Mrd.** **€** Förderung, u. a. KfW-Programm für Ladeinfrastruktur^b^, Einrichtung eines „Zukunftsfonds Automobilindustrie“, Verlängerung der Innovationsprämie bis 2025, nationales Flottenerneuerungsprogramm, Aufstockung Programme Konjunkturpaket, gesetzliche Vorgaben zur Einrichtung von Ladestationen (Bundesregierung 2020b)

*Quelle:* Eigene Zusammenstellung nach o. g. Quellen sowie den Stellungnahmen des VDA (Onlinematerial 1); Berechnung der Fördersummen nach Deutscher Bundestag, BT-Drs. 19/25739, Koalitionsausschuss (2020) und BMVI (2020c)

^a^Es ist unklar, inwieweit es sich um *neue* Förderzusagen handelt (BMVI 2020c, S. 6–7)

^b^Das Programm ist kein Ergebnis des Gipfels, sondern wurde bereits am 06.10.2020 angekündigt (BMVI 2020a). Inwieweit vorherige Mittelzusagen eingerechnet wurden, ist nicht gänzlich nachzuvollziehen (Deutscher Bundestag, BT-Drs. 19/25739)

Auch im weiteren Verlauf des Jahres 2020 ist der Automobilkorporatismus deutlich sichtbar (s. Tab. 2): Es folgten drei weitere Gipfeltreffen zwischen VDA, Automobilkonzernen, IG Metall, Ländern und Bundesregierung im Rahmen der bereits 2019 etablierten *‚Konzertierten Aktion Mobilität‘* (Deutscher Bundestag, BT-Drs. 19/11025; Deutscher Bundestag, BT-Drs. 19/25739) sowie auf dem dezidierten *‚Nutzfahrzeuggipfel‘* (BMVI 2020b). Die Automobilindustrie war hier nicht nur eng eingebunden, sondern, sowohl mit Blick auf die zeitliche Sequenz als auch die inhaltliche Kongruenz von Verbandspositionen und Beschlüssen, sehr erfolgreich darin, Unterstützung zu generieren. So fokussierte der VDA über Sommer und Herbst hinweg insbesondere auf die weitere Förderung der E‑Mobilität und der Ladeinfrastruktur (VDA u. a., 08.07.2020; VDA, 21.09.2020, 23.10.2020, 28.10.2020, 13.11.2020) oder forderte ein Unterstützungsprogramm für mittelständische Zulieferbetriebe (VDA, 24.09.2020). Während der VDA bis zum Frühherbst zwar keine messbaren finanziellen Zusagen erhielt (s. Autogipfel v. 08.09.2020), besteht sein ‚Erfolg‘ insgesamt darin, Themen strategisch zu platzieren und Beschlüsse über mehrere ‚Autogipfel‘ hinweg vorzubereiten (z. B. Ladeinfrastruktur). Seine Durchsetzungsfähigkeit ist bei einem Blick auf die Ergebnisse der Automobilgipfel des *gesamten* zweiten Halbjahres 2020 dann letztlich auch finanziell quantifizierbar (s. Tab. 2): Zwischen September und Dezember 2020 wurden weitere 3,2 Mrd. € zugesagt, die der Automobilindustrie direkt oder indirekt zugutekommen, z. B. die Flottenmodernisierung von öffentlicher Hand und Unternehmen (1 Mrd. €) oder die Einrichtung eines ‚Zukunftsfonds Automobilindustrie‘ (1 Mrd. €) mit einem Fokus auf die mittelständischen Zulieferbetriebe (Deutscher Bundestag, BT-Drs. 19/25739). Und auch einzelne Maßnahmen aus dem Konjunkturpaket wurden weiter aufgestockt, z. B. die Innovationsprämie (+ 1 Mrd. € bis 2025). Weitere rd. 4 Mrd. € konnten auf dem Nutzfahrzeuggipfel verbucht werden.

Mit allen Maßnahmen ist hierbei auch im Sinne des wechselseitigen Nutzens von organisierten Interessen und Regierung eine Verpflichtung der Automobilindustrie verbunden, die sich jedoch vom vorherigen Automobilkorporatismus unterscheidet. Der ‚Tausch‘ zwischen organisierten Interessen und Regierung fokussiert jetzt nicht länger nur auf die Arbeitsplatzsicherung (in der Automobil- wie in der Zulieferindustrie) gegen die Verabschiedung von Förderprogrammen, sondern beinhaltet eine dezidiert strukturpolitische Komponente; nämlich die Erwartung der Regierung, dass die Automobilhersteller ihre Produkte mittelfristig im Sinne klimapolitischer Vorgaben erneuern (während die Regierung die Ladeinfrastruktur fördert und bereitstellt). Obwohl unsere Befunde also insgesamt auf die Persistenz des Automobilkorporatismus hinweisen, zeigt sich auch eine graduelle Veränderung in der Coronakrise; nämlich im Sinne eines grün gefärbten ‚Korporatismus ohne Verbrennungsmotor‘ (Sack et al. 2021).

### Phase 3: Zustimmung, Resignation und Ablehnung

#### Krisenkorporatismus in der Krise?

Die Übereinstimmung von Bundesregierung und Wirtschaftsverbänden ließ mit Beginn der Diskussionen um einen erneuten (Teil‑)Lockdown ab Oktober 2020 deutlich nach. Dies gilt zugleich für die korporatistische Handlungskoordination: Die Verbände waren ganz offenkundig nicht mehr derart eng in die Krisenbewältigung eingebunden wie in der Frühphase der Pandemie. An die Stelle ‚korporatistischen‘ Kompromisses und Tauschs trat die exekutive Entscheidung zu restriktiven Infektionsschutzmaßnahmen in Bund-Länder-Konferenzen, die (aus Sicht der Verbände) nur teils kompensiert wurden (Überbrückungshilfen) und welche die Verbände wiederum öffentlich kritisierten.

Die Positionen der Verbände unterschieden sich *im Vorfeld* des ersten (Teil‑)Lockdowns^17^ nur graduell. So wurde gefordert, einen „erneuten Shutdown [zu] vermeiden“ (Börner, Präs. BGA in FAZ.net 2020e) oder vor der Gefahr „immer neuer unbegründeter Verbote“ (Zöllick, Präs. DEHOGA, 27.10.2020) gewarnt. Sichtbar wird, dass zwar einerseits die unmittelbar betroffenen Branchen deutlich offensiver auftraten (Einzelhandel, Tourismus, Gastronomie), andererseits aber innerhalb der Wirtschaft das Verständnis davon vorherrschte, dass alle Bereiche eng miteinander verzahnt sind, wie etwa Vertreter der Industrie argumentierten, z. B. der VDMA (FAZ.net 2020c). Bereits im Vorfeld wurde zudem der Ruf nach Kompensationen und Hilfsgeldern im Falle erneuter Geschäftsschließungen laut (F.A.Z. 2020a). Nach Beschluss über einen (Teil‑)Lockdown und dessen Inkrafttreten im November 2020 zeigten sich dann deutlichere Unterschiede zwischen Branchen und Wirtschaftsbereichen, die sich vor allem aber nicht nur an der unmittelbaren Betroffenheit von den Infektionsschutzmaßnahmen ausdifferenzieren. Hinsichtlich der Inhalte der öffentlichen Forderungen lassen sich drei Gruppen von Verbänden im Kontinuum von Zustimmung bis fundamentale Ablehnung identifizieren (Tab. 3). So wurden die Maßnahmen entweder *grundsätzlich als verhältnismäßig akzeptiert*, nach außen *‚resigniert‘ hingenommen* oder es wurde *grundsätzlich das bis dahin unter den Verbänden (öffentlich) weitgehend akzeptierte Primat des Infektionsschutzes infrage gestellt*. Hinter diesen inhaltlichen Ausprägungen lassen sich auch dezidierte Lobbying-Strategien einzelner Verbände erkennen.

**Table 3:** 

Gruppe	‚Zustimmung‘	‚Resignation‘	‚Ablehnung‘
Inhaltliche Position	**Überwiegende** Befürwortung der Maßnahmen bei Forderung nach Entlastungen	Hinnahme der Maßnahmen bei **deutlicher Kritik**, Forderung nach langfristiger Strategie, Planbarkeit	**Überwiegende** Ablehnung der Maßnahmen, Infragestellung der Verhältnismäßigkeit
Zugeordnete Wirtschaftsverbände^a^	VDMA *(Maschinenbau)* ZDH *(Handwerk)* ZGV *(Mittelstand)*	DEHOGA *(Hotels/Gastro)* HDE *(Einzelhandel)* FamUnt *(Familienuntern.)* BDA *(Arbeitgeber)* BDI *(Industrie)* bevh *(E-Commerce)*^*b*^	BDWI *(Dienstleistungen)* BVDW *(Mittelstand)* BGA *(Groß‑/Außenhand.)* BTW *(Tourismus)*

*Quelle:* Eigene Zusammenstellung nach Auswertung von Verbandsstellungnahmen (Onlinematerial 1)

^a^Es werden nur Verbände aufgeführt, die in diesem Zeitraum einschlägige Stellungnahmen und Positionen veröffentlichten

^b^Der bevh organisiert auch ‚Multichannel‘-Händler, die von Geschäftsschließungen betroffen waren

Nur wenige Verbände bewerteten die Infektionsschutzmaßnahmen im November und Dezember 2020 trotz ihrer Kritik als grundlegend positiv, vollumfänglich notwendig und verhältnismäßig. So betonte u. a. der *Zentralverband des Deutschen Handwerks (ZDH)* die Notwendigkeit des Infektionsschutzes, auch für die Funktionsfähigkeit der (Handwerks‑)Betriebe: „Persönlicher Gesundheitsschutz ist insofern immer auch Betriebe-Schutz“ (Wollseifer, Präs. ZDH, 25.11.2020). Dies galt auch für den VDMA, der die Maßnahmen als „richtig und notwendig, um die Ansteckungswelle zu brechen“ bezeichnete (Haeusgen, Präs. VDMA, 29.10.2020). Auffällig ist, dass es sich um Vertreter solcher Wirtschaftsbereiche handelt, die nicht unmittelbar von Schließungen und Einschränkungen (in größerem Umfang) betroffen waren. Allerdings war auch unter diesen Verbänden die Haltung zu den politischen Beschlüssen kritisch, z. B. mit Blick auf fehlende Planungsperspektiven oder den Umfang von Hilfsmaßnahmen. So forderte z. B. der BDI trotz Befürwortung der Maßnahmen „das Risiko von Jo-Jo-Shutdowns für Wirtschaft und Gesellschaft zu reduzieren“ (Kempf, Präs. BDI in FAZ.net 2020e).

Die Bewertung der Maßnahmen fiel durch Vertreter stark betroffener Branchen wie dem Einzelhandel oder der Gastronomie erwartungsgemäß deutlich kritischer aus, wenn auch die Notwendigkeit von tiefgreifenden Maßnahmen nicht infrage gestellt wurde. Insgesamt schien es im Sinne einer Lobbying-Strategie das Ziel zu sein, ‚Schlimmeres zu verhindern‘, während die Beschlüsse nach außen ‚resignierend‘ zur Kenntnis genommen wurden. So rückten Forderungen nach umfassenden und schnellen Hilfsmaßnahmen oder technische Aspekte in den Mittelpunkt der Verbandspositionen. Der *Handelsverband Deutschland (HDE)* konzentrierte sich z. B. auf Detailforderungen wie nach einer Vergrößerung der zulässigen Kundenzahl/Verkaufsfläche oder der genauen Umsetzung der ‚November- und Dezemberhilfen‘ (HDE, 28.10.2020a/b, 05.11.2020, 26.11.2020). Besonders eindrücklich ist die ‚Resignations‘-Strategie jedoch im Fall des *Deutschen Hotel- und Gaststättenverbands (DEHOGA) *nachzuvollziehen. Dieser war in der *unmittelbaren* Reaktion auf den erneuten Lockdown dezidiert kritisch und stellte Verhältnismäßigkeit wie Sinnhaftigkeit der Maßnahmen infrage; auch unter Androhung von Klagen (Hartges, Hauptgf. DEHOGA in F.A.Z. 2020a; FAZ.net 2020d). Nach dieser anfänglichen und öffentlichkeitswirksamen ‚Entrüstung‘ fokussierte der Verband jedoch – analog zum HDE – in deutlich konzilianterem Ton auf die schnelle Umsetzung von Hilfsmaßnahmen (F.A.Z. 2020b), „um das Vertrauen von Unternehmern in das gesprochene Wort der Politik“ (Zöllick, Präs. DEHOGA, 03.11.2020) zu erhalten. Insgesamt wurden im November und Dezember durch den DEHOGA nur noch (scheinbare) Rückzugsgefechte geführt und Detailfragen adressiert, als z. B. längere Öffnungsphasen für Hotels, statt kurzer Öffnungen über die Weihnachtsfeiertage gefordert wurden (F.A.Z. 2020c). Hierbei beinhaltete die offensive Reaktion eine Botschaft an die eigenen Mitglieder, während der Verband in Zurückhaltung und Resignation zugleich anschlussfähig für die weitere Interessenabstimmung mit der Bundesregierung blieb – und sich als ‚zuverlässiger Partner‘ eben nicht in die Fundamentalopposition gegen die Maßnahmen begab.^18^

Eine dritte Gruppe von Wirtschaftsverbänden trat deutlich offensiver und teils polemisch gegen die Maßnahmen an. Hier überwog die grundsätzliche Infragestellung des bisherigen Konsenses (Primat des Infektionsschutzes) und der Verhältnismäßigkeit der Maßnahmen. Als Vertreter einer stark betroffenen Branche wird dies für den *Bundesverbands der Deutschen Tourismuswirtschaft (BTW) *deutlich, der die Branche auf dem Weg zum „dauerhaften willkürlichen Spielball der Coronapolitik“ (Rabe, Generalsekr. BTW, 25.11.2020) sah. Es wurde sodann eine neue Abwägung, nämlich jene der „Balance zwischen gesundheitlicher Prävention und volkswirtschaftlicher Prosperität“ (Rabe, BTW, 29.10.2020), vorgenommen und die „Sinnhaftigkeit [der Maßnahmen] bei der Bekämpfung der Virusverbreitung“ hinterfragt (Rabe, BTW, 29.10.2020). Kritisiert wurde zudem – und dies unterstreicht den Befund fehlender Abstimmung von Wirtschaftsverbänden und Politik – die mangelnde Einbindung der Wirtschaft in die Planung der Maßnahmen (BTW, 25.11.2020). Noch deutlicher wird die ablehnende Haltung im Fall des *Bundesverbands mittelständische Wirtschaft (BVMW)*. Dieser war bereits seit August 2020 offensiv gegen die Regierungspolitik angetreten, u. a. mit einem ‚Brandbrief‘ an die Politik, in dem vor dem „Schreckgespenst eines zweiten Lockdown[s]“ als „Todesstoß“ für viele mittelständische Unternehmen gewarnt, Massenarbeitslosigkeit prognostiziert und „überzogener Infektionsschutz“ kritisiert wurde (BVMW, 25.08.2020). Im Mittelpunkt standen dann ab Herbst Zweifel an der Verhältnismäßigkeit der Maßnahmen und deren politischer („am Bundestag vorbei“) wie rechtlicher („[fraglich ob] im Einklang mit unserer Verfassung“) Legitimation (Ohoven, Präs. BVMW, 29.10.2020).

Insgesamt kann für Oktober bis Dezember 2020 eine zunehmende Distanz zwischen Wirtschaftsverbänden und Bundesregierung konstatiert werden. Diese lässt sich an der Verlagerung der Auseinandersetzung in die Medien, strategischem Lobbying und der inhaltlichen Distanzierung des Gros der Wirtschaftsverbände festmachen. Dem prägenden krisenkorporatistischen Tausch – Einbeziehung und Berücksichtigung der Verbandsforderungen gegen öffentliche Unterstützung und Implementation der Hygieneschutzmaßnahmen – wurde in dieser Phase zumindest in Teilen die Grundlage entzogen – der Krisenkorporatismus pausierte.

#### Gute Vorsätze?

Allerdings folgte mit Beginn des Jahres 2021 der verbands- wie regierungsseitige Versuch, korporatistische Handlungskoordination wiederzubeleben. Der ‚Wirtschaftsgipfel‘ von Bundeswirtschaftsminister Altmaier mit rund 40 Wirtschaftsverbänden^19^ (BMWE 2021) markiert hier eine wichtige Schlüsselsituation.

Im Vorfeld des Gipfels verschärfte sich jedoch zunächst die Kritik der Verbände. Es wurden einhellig Lockerungen und ein „Wiederöffnungsplan“ (FamUnt, 08.02.2021) sowie klare, strategische und regelbasierte Entscheidungen über ökonomische Einschränkungen mit bundesweiter Gültigkeit gefordert (FAZ.net 2021a; F.A.Z. 2021a, b). Auch bisherige Befürworter der Maßnahmen wurden deutlicher in ihrer Kritik. Anschaulich lässt sich dieser (graduelle) Wandel in den Positionen am ZDH nachvollziehen, der noch Ende 2020 zu den wenigen expliziten Unterstützern der Maßnahmen zählte – und *Infektionsschutz „immer auch [als] Betriebe-Schutz“* verstand (s. oben). Nun ging es dem ZDH hingegen um einen „transparenten und regelbasierten Öffnungsplan[.]“, der „*nicht einseitig den Infektionsschutz *in den Blick nimmt, sondern […] *zwingend auch den Betriebe- und Wirtschaftsschutz*“ (Wollseifer, Präs. ZDH, 16.02.2021, eig. Hervorh.). Das Primat des Infektionsschutzes (für das ggf. Kompensationen gefordert wurden) wurde damit nicht länger akzeptiert, sondern eine neue Güterabwägung zwischen Gesundheit und wirtschaftlichen Interessen vorgenommen. Die bisherigen Kritiker formulierten zugespitzter: So sprach der HDE nach einer weiteren Verlängerung des Lockdowns von „Wortbruch“ und „Willkür“ (Gent, Geschf. HDE in FAZ.net 2021b), der BVMW vom „Gipfel der Enttäuschungen für den Mittelstand“ (FAZ.net 2021c) und der BTW sah seine Mitgliedsunternehmen als „wirtschaftliche Intensivpatienten dieser Pandemie“, die durch die Politik ‚stigmatisiert‘ würden (Frenzel, Präs. BTW, 16.02.2021).

Auffällig ist aber nicht nur, dass die Positionen deutlich kritischer wurden, sondern dass sich die Wirtschaftsverbände wie in der Frühphase der Krise wieder stärker mit eigenen Konzepten und Vorschlägen zu Wort meldeten. Es erfolgte der verbandsseitige Versuch einer Revitalisierung korporatistischer Handlungskoordination: So unterbreitete z. B. der BDI erneut eigene Vorschläge für eine wirtschaftspolitische Strategie (BDI, 16.02.2021). Die BDA wiederum schlug eine „Konzertierte[.] Aktion von Gewerkschaften und Arbeitgebern“ vor, um die Regierung zu unterstützen (Dulger, Präs. BDA, 16.02.2021). Insgesamt kann also nicht von einem Kooperationsentzug der Wirtschaftsverbände gesprochen werden, sondern vielmehr von artikulierter Unzufriedenheit bei gleichzeitigen Angeboten, an einer Veränderung der Situation – in ihrem Sinne – aktiv mitzuwirken.

Der Wirtschaftsgipfel (16.02.2021) ist als Reaktion der Bundesregierung auf diese artikulierte Unzufriedenheit wie auch auf das Kooperationsangebot zu verstehen. Er brachte jedoch nur wenige konkrete Ergebnisse, abgesehen vom Versprechen einer schnelleren Auszahlung der Hilfsgelder bzw. deren Ausweitung (Süddeutsche.de 2021).^20^ Dementsprechend kritisch fiel die Bewertung der Verbände im Nachlauf des Gipfels aus; sie beklagten weiterhin die fehlende Langfriststrategie und eine langsame und bürokratische Bearbeitung der Wirtschaftshilfen (F.A.Z. 2021b, c). Mit Blick auf die Praktiken der Interessenintermediation war der Gipfel *an sich* jedoch Zeichen eines graduellen Wandels. Dies zeigt sich auch daran, dass die Wirtschaftsverbände zu Vorschlägen für eine (Öffnungs‑)Strategie aufgefordert wurden (FAZ.net 2021d; F.A.Z. 2021c), die das BMWE sodann Anfang März in die Bund-Länder-Abstimmung einbrachte (ZEIT Online 2021). Allerdings bleibt unklar, ob die Vorschläge tatsächlich bei der weiteren Planung der Infektionsschutzmaßnahmen eine Rolle spielten oder im Prozess der Regierungsabstimmung ‚verpufften‘.

## Konklusion

Zwei Fragen standen im Mittelpunkt unseres Beitrags: Ob und wie artikulieren sich organisierte Wirtschaftsinteressen in der Coronakrise? Nimmt die Interaktion zwischen Regierung und Wirtschaftsverbänden den Charakter eines „Krisenkorporatismus“ (Rehder 2021) an? Wir haben sowohl die Einbindung deutscher Arbeitgeberverbände, Wirtschaftsverbände und -kammern in die Aushandlung der (ökonomischen) Krisenbewältigungsstrategien als auch ihren Beitrag zur Umsetzung der Wirtschaftspolitik im Sinne korporatistischer Handlungskoordination untersucht. Unsere eingehende empirische Validierung bestätigt erste Beobachtungen zum „Krisenkorporatismus“ (Rehder 2021) im von uns fokussierten ersten Jahr der Coronakrise (März 2020–Februar 2021). Der ‚Krisenkorporatismus 2020/21‘ schließt an (bereits zuvor in Veränderung befindlichen) Traditionen korporatistischer Praktiken an. Es zeigt sich also – und wir beziehen uns hier auf den Ausschnitt der benannten Krisensituation – ein hohes Maß an Kontinuität, Stabilität und Pfadabhängigkeit (Pierson 2004). Die untersuchungsleitenden Hypothesen zur pfadabhängigen Revitalisierung korporatistischer Handlungskoordination (H1) und der Einbeziehung der Wirtschaftsverbände als Ressource (H2) werden in diesem Sinn bestätigt. Es sind insbesondere große und ressourcenstarke Verbände, die kontinuierlich in die Krisenbewältigung einbezogen wurden und werden. Die Interaktion zwischen Staat und Verbänden orientiert sich pfadabhängig an etablierten und eingespielten Kooperationsformen. Wirtschaftsgipfel und Verbändegespräche binden solche Akteure ein, die bereits vorher wesentliche Ansprechpartner der Regierung waren, bestehende Formate werden genutzt, um die Krisenpolitik abzustimmen. Es zeigen sich jedoch auch Besonderheiten: Die Staat-Verbände-Interaktion ist – im Gegensatz zur klassischen korporatistischen Konzertierung – durch die auch öffentlich nachvollziehbare Abstimmung mit einem großen Teilnehmerfeld gekennzeichnet. Gründe für diese Öffentlichkeit wie für die ‚Pluralität‘ des krisenkorporatistischen Arrangements sehen wir in der Beschaffenheit der Coronakrise als umfassende ökonomische wie gesellschaftliche Problemstellung einerseits und der inhaltlich spezifischen sowie in ihrem Ausmaß differenten Betroffenheit einzelner Wirtschaftsbereiche andererseits. Allerdings ist der Krisenkorporatismus in 2020/21 durch ausgeprägte Konjunkturen gekennzeichnet: Es ist vor allem die erste Phase der akuten Krisenbewältigung, in der organisierte Wirtschaftsinteressen eng in die wirtschaftspolitische Bewältigung der Krise eingebunden sind. Ihre Aktivierung als Ressource – sei es durch die umfassende Information der Mitgliedsunternehmen oder die Bereitstellung von Dienstleistungen zur Bewältigung der Krise – wird gegen die Berücksichtigung ihrer Vorschläge bei der Ausgestaltung wirtschaftspolitischer Maßnahmen ‚getauscht‘. Im weiteren Verlauf, insbesondere im Herbst 2020, zeigt sich jedoch, dass die Kooperationsbereitschaft organisierter Interessen – ganz im Sinne der korporatistischen ‚Tauschlogik‘ (Lehmbruch 1978, 1983) – immer auch von ihrer kontinuierlichen Einbindung und der Berücksichtigung ihrer Forderungen abhängt; letzten Endes sind Wirtschaftsverbände wie -kammern Interessenvertreterinnen und Repräsentantinnen der (sie finanzierenden und unterstützenden) Mitgliedsunternehmen. Auffällig ist hier, dass es im Kontinuum von Unterstützung bis Ablehnung der politischen Maßnahmen eine nennenswerte Zahl von Verbänden gibt, die sich ‚resignierend‘ nur noch technischen Details und Kompensationsfragen widmen, ohne substantielle eigene Vorschläge einzubringen. Wir sehen hierin eine dezidierte Strategie, um abseits der öffentlichen Diskussion – und damit für uns hier nicht einsehbar – weiterhin einbezogen zu werden. Dies ändert sich Anfang 2021 mit Vorschlägen für konkrete ‚Öffnungsstrategien‘. Hier deutet der *Versuch* von Politik wie von Verbänden, eine Revitalisierung enger Abstimmung und der Einbindung der Wirtschaftsverbände zu erreichen, wiederum auf die Persistenz korporatistischer Praktiken hin. Wir kommen daher zu dem Ergebnis, dass die Zwischenphase der merklichen Distanzierung der organisierten Interessen von der Politik der Bundesregierung keiner grundlegenden Abkehr vom Krisenkorporatismus geschuldet ist.

Zeichen der graduellen Veränderung zeigen sich damit nur am Rande. Veränderungen finden sich, wie am Beispiel der Automobilindustrie aufgezeigt wurde, vor allem auf inhaltlicher Ebene und in der Frage, wie Ergebnisse korporatistischer Politikverhandlung öffentlich kommuniziert werden. Es geht hier keineswegs um einen fundamentalen Wandel korporatistischer Praktiken, sondern um leichte Anpassungen – im Fall der Automobilindustrie sprechen wir von einem ‚Korporatismus ohne Verbrennungsmotor‘ (Sack et al. 2021) Beobachtungen in der fachwissenschaftlichen Debatte (von Winter und Willems 2007; Rehder und van Elten 2019), dass es anhaltende und tiefgreifende Veränderungen des Korporatismus insgesamt gibt, lassen sich für das Krisenjahr 2020/21 nicht bestätigen. Anders ausgedrückt: Krise revitalisiert Korporatismus. Als reproduktive Mechanismen der pfadabhängigen Interaktionsmuster (Pierson 2004) lassen sich einerseits der Ressourcentausch zwischen Wirtschaftsverbänden und Regierung und anderseits die bestehenden formellen Netzwerke identifizieren.

Unsere Ergebnisse können somit als Ausgangspunkt einer weiterführenden fachwissenschaftlichen Debatte dienen. Dahingehend steht die Interessengruppenforschung, sowohl mit dem Blick nach vorne als auch zurück in die Geschichte der unterschiedlichen „Konjunkturen des Korporatismus“ (Czada 1994), vor der Aufgabe, die beschriebenen krisensituativen Interaktionen zwischen Staat und Verbänden weiter empirisch zu prüfen und konzeptionell zu diskutieren.

## Supplementary Information





## Footnotes

1 Aus Platzgründen, insbesondere zugunsten der umfassenden Darstellung der Fallstudienergebnisse, finden sich ausführliche Hinweise zu Methodik und Datengrundlage in Onlinematerial 1.

2 Junk et al. (2021) befassen sich in einer international vergleichenden Studie zuvorderst mit dem Zugang von Interessengruppen zu Medien, Parlamenten, Regierungen und Bürokratie in der Coronakrise. Komplementär dazu konzentrieren wir uns auf die Interaktionen und programmatischen Kongruenzen zwischen Staat und Wirtschaftsverbänden in Deutschland in der Krise.

3 Hiermit ist auch verbunden, dass wir uns explizit nicht dem tripartiten ‚Makrokorporatismus‘ zwischen Staat, Wirtschaftsverbänden und Gewerkschaften widmen können.

4 Unsere Auswertung erfasst *öffentlich nachvollziehbare *Interaktionen. Die Reichweite unserer Analyse ist insoweit selbstredend begrenzt. Denn natürlich finden weitere Kontakte auf der politischen ‚Hinterbühne‘ statt, z. B. in wöchentlichen „Verbände-Telefonkonferenz[en]“ des Bundeswirtschaftsministeriums (Deutscher Bundestag, BT-Drs. 19/21363). Und ebenso gibt es die üblichen Verbändeanhörungen im Gesetzgebungsprozess (§ 47 GGO der Bundesministerien und § 70 GO des Bundestages).

5 Die regelmäßigen Teilnehmerverbände der Treffen im Kanzleramt finden sich in Onlinematerial 1.

6 Zudem wird der etablierte ‚Mittelstandsbeirat‘ des BMWE, der sich aber nicht aus Verbänden, sondern aus Einzelpersonen zusammensetzt, mehrfach konsultiert (Deutscher Bundestag, BT-Drs. 19/22073).

7 Diese ist auch unter Berücksichtigung der saisonal bedingten ‚Ruhe‘ in Unternehmen, Verbänden und Politik, die mit Vorweihnachtszeit und Jahreswechsel einhergeht, auffällig.

8 Zur Anlage und Methode der Auswertung der Verbandsaktivitäten siehe Fuchs et al. (2021).

9 Als Beitrag der Wirtschaftsverbände und -kammern zur Implementation der Infektionsschutzmaßnahmen können u. a. die Beratung und ‚Verpflichtung‘ der Mitglieder auf die Umsetzung von Hygienekonzepten (v. a. Handel und Dienstleistungen), die Umsetzung des Infektionsschutzes in Betrieben (inkl. der Ermöglichung von Homeoffice) oder die Beratung bei und die Koordinierung von Produktionsumstellungen (Masken, Desinfektionsmittel) verstanden werden.

10 Diese Auswahl deckt eine große Zahl an Wirtschaftsbereichen ab (Kohler-Koch et al. 2021). So ist die AGM ein Zusammenschluss zahlreicher Einzelverbände, u. a. des *Zentralverbands des Deutschen Handwerks* (ZDH) sowie von Verbänden des Mittelstands (ZGV), der Freien Berufe (BfB), von Touristik- und Gastwerbe (DRV, DEHOGA), Handels- (BGA, HDE) und Bankenverbänden (BVR, SPKV). Die *Bundesvereinigung Deutscher Arbeitgeberverbände* (BDA) als weiterer Spitzenverband formulierte keine *öffentlich* nachvollziehbaren Forderungen im Vorlauf der Verabschiedung des Konjunkturpakets.

11 Eine Liste der nachfolgend referenzierten Verbandsstellungnahmen (Akronym, Datum) findet sich in Onlinematerial 1.

12 Auf mögliche Gründe für Unterschiede in den Positionen der Verbände bzw. Verbändezusammenschlüsse können wir hier aus Platzgründen nicht eingehen.

13 Weitere nachfrageorientierte Maßnahmen wurden von Einzelverbänden außerhalb ihrer Beteiligung an den Spitzenverbandspositionen gefordert (und umgesetzt) – so die vom *Bundesverband der Deutschen Volksbanken und Raiffeisenbanken (BVR) *geforderte „vorübergehende Absenkung des Mehrwertsteuersatzes“ (BVR, 29.05.2020).

14 Der VDA ist – ebenso wie Verbände von Zulieferindustrien – Mitglied des BDI.

15 Einbezogen in die Berechnung wurden folgende Posten des Konjunkturpakets (Koalitionsausschuss 2020): 35b (Innovationsprämie, 2,2 Mrd. €), 35c (Bonusprogramm Zukunftsinvestitionen, 2 Mrd. €), 35d (Flottenaustauschprogramm Soziale Dienste, 200 Mio. €), 35f (Investitionen Ladeinfrastruktur E‑Mobilität, 2,5 Mrd. €) und 35i (Flottenerneuerung Busse und LKW, 1,2 Mrd. €). Nicht berücksichtigt wurde der Posten 35e (Flottenaustauschprogramm für Handwerker, 30 Mio. €), da nicht ersichtlich ist, ob es sich um zusätzliche Finanzmittel oder die Umwidmung bestehender Fördermittel (Nutzfahrzeuge) handelt (Deutscher Bundestag, BT-Drs. 19/25739, S. 10).

16 Hinzu kommen weitere Maßnahmen, von denen die Automobilindustrie mittelbar profitiert, z. B. die nationale Wasserstoffstrategie oder die fortgesetzte KfZ-Steuer-Befreiung für E‑Fahrzeuge (Deutscher Bundestag, BT-Drs. 19/25739, S. 9–13).

17 Für die Positionierung der Wirtschaftsverbände sind drei Zeitpunkte relevant, nämlich (1) der Beschluss zu einem Teil-Lockdown am 28.10.2020 (Inkrafttreten: 03.11.2020), (2) der Beschluss zu einer Verlängerung und Verschärfung des Lockdowns am 25.11.2020 (Inkrafttreten: 01.12.2020) sowie (3) der Beschluss für einen ‚harten‘ Lockdown am 13.12.2020 (Inkrafttreten: 16.12.2020).

18 Diese Strategie lässt sich bereits in der ersten Phase beobachten. Hier bewertet der DEHOGA gemeinsam mit anderen Tourismusverbänden (BTW u. a., 04.06.2020, s. oben) das Konjunkturpaket kritischer als in seiner Einzelstellungnahme (DEHOGA, 04.06.2020) und stimmte sich zugleich eng mit dem BMWE ab. Dies ist z. B. für regelmäßige Konsultationen zum (ersten/zweiten) Corona-Steuerhilfegesetz dokumentiert (Deutscher Bundestag, BT-Drs. 19/21363; Deutscher Bundestag, BT-Drs. 19/22363).

19 Eine genaue Teilnehmerliste liegt uns nicht vor. Aus unseren Recherchen ist jedoch ersichtlich, dass die hier fokussierten Verbände beteiligt waren.

20 Die Überbrückungshilfe III wurde – wie vom HDE dezidiert gefordert (15.02.2021) – auf Betriebe mit >750 Mio. € Jahresumsatz ausgeweitet.

## References

[CR1] Baumgartner FR, Berry JM, Hojnacki M, Kimball DC, Leech BL (2009). Lobbying and policy change. Who wins, who loses, and why.

[CR3] Behrens M (2011). Das Paradox der Arbeitgeberverbände. Von der Schwierigkeit, durchsetzungsstarke Unternehmensinteressen kollektiv zu vertreten.

[CR4] Behrens M, Schroeder W, Weßels B (2017). Strukturen der Interessenvertretung in den Verbänden der Wirtschaft. Handbuch Arbeitgeber- und Wirtschaftsverbände in Deutschland.

[CR7] BMVI – Bundesministerium für Verkehr und digitale Infrastruktur (2020). Gesamtkonzept klimafreundliche Nutzfahrzeuge, November 2020.

[CR5] BMVI – Bundesministerium für Verkehr und digitale Infrastruktur. 2020a. Infopapier zur Förderung von Ladesäulen, 6. Oktober 2020. https://www.bmvi.de/SharedDocs/DE/Anlage/Infopapierladesaulen.pdf?__blob=publicationFile. Zugegriffen: 28. Apr. 2021.

[CR6] BMVI – Bundesministerium für Verkehr und digitale Infrastruktur. 2020b. Nutzfahrzeuggipfel: Mit alternativen Antrieben auf dem Weg zur Nullemissionslogistik auf der Straße, 10. November 2020. https://www.bmvi.de/SharedDocs/DE/Video/Youtube/nutzfahrzeuggipfel-11-11-2020.html. Zugegriffen: 28. Apr. 2021.

[CR8] BMWE – Bundesministerium für Wirtschaft und Energie, und VDA – Verband der Automobilindustrie. 2020. Minister Altmaier und VDA-Präsidentin Müller würdigen Einsatz der Automobilindustrie in Corona-Krise. Pressemitteilung. 07. April 2020. https://www.bmwi.de/Redaktion/DE/Pressemitteilungen/2020/20200407...ler-wuerdigen-einsatz-der-automobilindustrie-in-corona-krise.html. Zugegriffen: 28. Apr. 2021.

[CR9] BMWE – Bundesministerium für Wirtschaft und Energie. 2020. Altmaier: „Wir wollen, dass der Wohlstand wieder wächst“. Arbeitsgruppe für konjunkturbelebende Maßnahmen tagt im BMWi. https://www.bmwi.de/Redaktion/DE/Pressemitteilungen/2020/20200514-altmaier-wir-wollen-dass-der-wohlstand-wieder-waechst.html. Zugegriffen: 25. März 2021.

[CR11] BMWE – Bundesministerium für Wirtschaft und Energie. 2021. Altmaier im Vorfeld des heutigen Wirtschaftsgipfels. Pressemitteilung. 16. Februar 2021. https://www.bmwi.de/Redaktion/DE/Pressemitteilungen/2021/02/20210216-altmaier-im-vorfeld-des-heutigen-wirtschaftsgipfels.html. Zugegriffen: 28. Apr. 2021.

[CR13] Bundesregierung. 2020a. Gestärkt aus der Krise, gemeinsam die Mobilität der Zukunft gestalten. 3. Spitzengespräch der Konzertierten Aktion Mobilität. Pressemitteilung 316 v. 08. September 2020. https://www.bundesregierung.de/breg-de/aktuelles/-gestaerkt-aus-den-3-spitzengespraech-der-konzertierten-aktion-mobilitaet-1783382. Zugegriffen: 25. März 2021.

[CR14] Bundesregierung. 2020b. 4. Spitzengespräch der Konzertierten Aktion Mobilität – „Transformation unterstützen, Wertschöpfungsketten stärken“. Pressmitteilung 410 v. 17. November 2020. https://www.bundesregierung.de/breg-de/aktuelles/4-spitzengespraech-der-konzertierten-aktion-mobilitaet-transformation-unterstuetzen-wertschoepfungsketten-staerken--1815818. Zugegriffen: 25. März 2021.

[CR16] Cross JP, Eising R, Hermansson H, Spohr F (2021). Business interests, public interests, and experts in parliamentary committees. Their impact on legislative amendments in the German Bundestag. West European Politics.

[CR17] Czada R (1994). Konjunkturen des Korporatismus: Zur Geschichte eines Paradigmenwechsels in der Verbändeforschung. Politische Vierteljahresschrift, Sonderheft.

[CR18] Deutscher Bundestag, Drucksache 19/11025. Antwort der Bundesregierung auf die Kleine Anfrage der Abgeordneten Cem Özdemir, Stephan Kühn (Dresden), Oliver Krischer, weiterer Abgeordneter und der Fraktion BÜNDNIS 90/DIE GRÜNEN – Drucksache 19/10473. Ziele, Beginn und Zeitrahmen der „Konzertierten Aktion Mobilität“, 21. Juni 2019.

[CR19] Deutscher Bundestag, Drucksache 19/21363. Antwort der Bundesregierung auf die Kleine Anfrage der Abgeordneten Jan Korte, Dr. Petra Sitte, Friedrich Straetmanns, weiterer Abgeordneter und der Fraktion DIE LINKE – Drucksache 19/20275. Einflussnahme von Interessenvertreterinnen und Interessenvertretern auf den Gesetzentwurf der Bundesregierung – Entwurf eines Gesetzes zur Umsetzung steuerlicher Hilfsmaßnahmen zur Bewältigung der Corona-Krise (Corona-Steuerhilfegesetz) (Bundesratsdrucksache 221/20), 30. Juli 2020.

[CR20] Deutscher Bundestag, Drucksache 19/22073. Antwort der Bundesregierung auf die Kleine Anfrage der Abgeordneten Claudia Müller, Erhard Grundl, Anja Hajduk, weiterer Abgeordneter und der Fraktion BÜNDNIS 90/DIE GRÜNEN – Drucksache 19/21467. Entstehung der Hilfen für Soloselbständige in der Corona-Pandemie, 02. September 2020.

[CR21] Deutscher Bundestag, Drucksache 19/22363. Antwort der Bundesregierung auf die Kleine Anfrage der Abgeordneten Jan Korte, Dr. Petra Sitte, Friedrich Straetmanns, weiterer Abgeordneter und der Fraktion DIE LINKE – Drucksache 19/21601. Einflussnahme von Interessenvertreterinnen und Interessenvertretern auf den Gesetzentwurf der Bundesregierung – Entwurf eines Zweiten Gesetzes zur Umsetzung steuerlicher Hilfsmaßnahmen zur Bewältigung der Corona-Krise (Zweites Corona-Steuerhilfegesetz) (Bundestagsdrucksache 19/20058), 14. September 2020.

[CR22] Deutscher Bundestag, Drucksache 19/25739. Antwort der Bundesregierung auf die Kleine Anfrage der Abgeordneten Oliver Luksic, Frank Sitta, Bernd Reuther, weiterer Abgeordneter und der Fraktion der FDP – Drucksache 19/25329. Maßnahmen und Ergebnisse der bisherigen „Autogipfel“, 08. Januar 2021.

[CR23] DLF. 2020. VDA-Präsidentin: Verbraucher motivieren, Pkw und Nutzfahrzeuge zu kaufen. Hildegard Müller im Gespräch mit Tobias Armbrüster, 29. April 2020. https://www.deutschlandfunk.de/kaufpraemien-fuer-automobilbranche-vda-praesidentin.694.de.html?dram:article_id=475685. Zugegriffen: 25. März 2021.

[CR24] Döhler M (2020). Ministerialverwaltung und Interessengruppen. Neues und Vergessenes zu einem alten Thema. Zeitschrift für Politikwissenschaft.

[CR25] Dorn F, Fuest C, Neumeier F (2020). Nach dem großen Einbruch. Ein Konjunkturprogramm zur Stützung und Erholung der Wirtschaft. Ifo-Schnelldienst.

[CR26] Dür A, Marshall D, Bernhagen P (2019). The political influence of business in the European Union.

[CR27] Eichhorst W, Weishaupt JT (2013). Mit Neokorporatismus durch die Krise? Die Rolle des sozialen Dialogs in Deutschland, Österreich und der Schweiz. Zeitschrift für Sozialreform.

[CR28] Eising R, Spohr F (2017). The more, the merrier? Interest groups and legislative change in the public hearings of the German parliamentary committees. German Politics.

[CR29] Ellguth P, Kohaut S (2020). Tarifbindung und betriebliche Interessenvertretung. Aktuelle Ergebnisse aus dem IAB-Betriebspanel 2019. WSI-Mitteilungen.

[CR91] van Elten K (2018). Profession und Selbstverwaltung. Die Legitimationspolitik von Wirtschafts- und Berufskammern.

[CR30] F.A.Z. (2020). Pläne für milden Lockdown. Die Bundesregierung erwägt, die Gastronomie zu schließen. Dagegen regt sich Widerstand, nicht nur von den Betroffenen. Von Corinna Budras und Julia Löhr, 28. Oktober 2020. Wirtschaft, Seite 15.

[CR31] F.A.Z. (2020). Geldregen soll Klagen verhindern. Der Staat zeigt sich beim zweiten Lockdown großzügiger. Das könnte bei den erwarteten Streitigkeiten helfen. Von Corinna Budras und Julia Löhr, 30. Oktober 2020. Wirtschaft, Seite 19.

[CR32] F.A.Z. (2020). Es ist ein furchtbares Durcheinander. Vor dem Treffen der Ministerpräsidenten mit der Kanzlerin kritisieren Gastronomen die neuen Pläne zum Lockdown für die Branche. Von Jacqueline Vogt, 25. November 2020. Rhein-Main-Zeitung, Seite 33.

[CR33] F.A.Z. (2021). Fahrplan für Öffnung gefordert. Wirtschaftsverbände zunehmend ungeduldig. 10. Februar 2021, Nr. 34, Seite 17.

[CR34] F.A.Z. (2021). Der Möchtegern-Kümmerer. Kaum Geld, kaum eine Perspektive: Die Kritik an Wirtschaftsminister Altmaier wächst. Ein Gipfel soll Abhilfe schaffen. Doch ein Problem bleibt. Von Julia Löhr, 16. Februar 2021, Nr. 39, Seite 17.

[CR35] F.A.Z. (2021). Die Wirtschaft ruft nach Merkel. Wirtschaftsminister Altmaier verspricht den geschlossenen Betrieben mehr finanzielle Hilfe und einen Öffnungsplan. Doch den Verbänden ist das nicht genug. Von Julia Löhr, 17. Februar 2021, Nr. 40, Seite 15.

[CR37] FAZ.net. 2020a. Regierung will bis Juni über Kaufprämien für Autos entscheiden. 05. Mai 2020. https://www.faz.net/aktuell/wirtschaft/regierung-will-bis-juni-ueber-kaufpraemien-fuer-autos-entschieden-16755149.html. Zugegriffen: 15. Apr. 2021.

[CR38] FAZ.net. 2020b. Geld nur für E‑Autos. Von Ilka Kopplin und Kerstin Schwenn, 04. Juni 2020. https://www.faz.net/aktuell/wirtschaft/auto-verkehr/corona-konjunkturpaket-der-bundesregierung-geld-nur-fuer-e-autos-16800836.html. Zugegriffen: 25. März 2021.

[CR40] FAZ.net. 2020c. Trotz Corona-Welle. Altmaier korrigiert Wachstumsprognose nach oben. Von Julia Löhr, 26. Oktober 2020. https://www.faz.net/aktuell/wirtschaft/konjunktur/altmaier-korrigiert-wachstumsprognose-nach-oben-17020593.html. Zugegriffen: 22. Apr. 2021.

[CR41] FAZ.net. 2020d. Merkels Mission für Weihnachten. Von Sebastian Reuter, 29. Oktober 2020. https://www.faz.net/aktuell/f-a-z-newsletter-merkels-corona-mission-fuer-weihnachten-17025136.html. Zugegriffen: 22. Apr. 2021.

[CR42] FAZ.net. 2020e. Corona-Pandemie. Kritik an Beschränkungen, Forderungen nach Entlastung. 26. November 2020. https://www.faz.net/aktuell/politik/inland/corona-kritik-an-beschraenkungen-und-forderungen-nach-entlastung-17071296.html. Zugegriffen: 22. Apr. 2021.

[CR43] FAZ.net. 2021a. Vor Bund-Länder-Gipfel. Wirtschaft fordert Lockerungen der Corona-Maßnahmen. 09. Februar 2021. https://www.faz.net/aktuell/wirtschaft/wirtschaft-fordert-lockerungen-der-corona-massnahmen-17188806.html. Zugegriffen: 8. Apr. 2021.

[CR44] FAZ.net. 2021b. Lockdown-Verlängerung. Handelsverband wirft Politik Wortbruch vor. 11. Februar 2021. https://www.faz.net/aktuell/wirtschaft/lockdown-verlaengerung-handelsverband-wirft-politik-wortbruch-vor-17192301.html. Zugegriffen: 8. Apr. 2021.

[CR45] FAZ.net. 2021c. Neue Corona-Beschlüsse. Scharfe Kritik an Plänen für Schulen und Handel. 11. Februar 2021. https://www.faz.net/aktuell/politik/inland/neue-corona-beschluesse-kritik-an-plaenen-fuer-schulen-und-handel-17191861.html. Zugegriffen: 8. Apr. 2021.

[CR46] FAZ.net. 2021d. Wirtschaftsgipfel mit Altmaier. „Ein völliges Wegducken ist nicht die Lösung“. Von Christoph Schäfer, 16. Februar 2021. https://www.faz.net/aktuell/wirtschaft/unternehmen/wirtschaft-und-corona-verbaende-fordern-strategie-fuer-oeffnungen-17200606.html. Zugegriffen: 8. Apr. 2021.

[CR47] Fuchs S, Sack D, Spilling F (2021). Function, shock or resources?—Organised business and the Covid-19 crisis in Germany.

[CR48] Grote JR, Lang A, Schneider V (2008). Organized business interests in changing environments. The complexity of adaptation.

[CR49] Grote JR, Lang A, Traxler F, Traxler F, Huemer G (2007). Germany. Handbook of business interest associations, firm size and governance.

[CR50] Haipeter T, Sack D, Strünck C (2016). Variety of Strategies. Arbeitgeberverbände ohne Tarifbindung in Deutschland. Verbände unter Druck. Protest, Opposition und Spaltung in Interessenorganisationen.

[CR51] Haipeter T, Schroeder W, Weßels B (2017). OT-Mitgliedschaften und OT-Verbände. Handbuch Arbeitgeber- und Wirtschaftsverbände in Deutschland.

[CR53] Junk WM, Crepaz M, Hanegraaff M, Berkhout J, Aizenberg E (2021). Changes in interest group access in times of crisis: no pain, no (lobby) gain. Journal of European Public Policy.

[CR54] Kluth W (2021). Das zweite DIHK-Urteil des Bundesverwaltungsgerichts und seine Folgen. NVwZ.

[CR56] Klüver H (2012). Die Macht der Informationen: Eine empirische Analyse von Lobbyingerfolg in der Europäischen Union. Politische Vierteljahresschrift.

[CR55] Klüver H, Zeidler E (2019). Explaining interest group density across economic sectors. Evidence from Germany. Political Studies.

[CR57] Koalitionsausschuss. 2020. Corona-Folgen bekämpfen, Wohlstand sichern, Zukunftsfähigkeit stärken. Ergebnis Koalitionsausschuss, 03. Juni 2020. https://www.bundesfinanzministerium.de/Content/DE/Standardartikel/Themen/Schlaglichter/Konjunkturpaket/2020-06-03-eckpunktepapier.pdf?__blob=publicationFile&v=12. Zugegriffen: 25. März 2020.

[CR58] Kohler-Koch B, Fuchs S, Friedrich DA (2021). Verbände mit Zukunft? Die Re-Organisation industrieller Interessen in Deutschland.

[CR59] Lehmbruch G (1978). Corporatism, Labour, and Public Policy. Konferenzbeitrag. Symposium “Social Policies in Comparative Perspective”. 9th World Congress of Sociology, August 1978. Uppsala.

[CR60] Lehmbruch G (1983). Neokorporatismus in Westeuropa: Hauptprobleme im internationalen Vergleich. Journal für Sozialforschung.

[CR61] Lehmbruch G, Windhoff-Héritier A (1987). Administrative Interessenvermittlung. Verwaltung und ihre Umwelt.

[CR62] Lesch H (2017). Mindestlohn und Tarifgeschehen. Die Sicht der Arbeitgeber in betroffenen Branchen.

[CR64] Miles MB, Huberman AM, Saldaña J (2014). Qualitative Data Analysis. A Methods Sourcebook.

[CR65] Pakull D, Goldberg F, Bernhagen P (2020). Die Repräsentation der Bürgerinnen und Bürger durch organisierte Interessen in Deutschland. Politische Vierteljahresschrift.

[CR66] Paster T (2015). Bringing power back in. A review of the literature on the role of business in welfare state politics.

[CR67] Pierson P (2004). Politics in time. History, institutions, and social analysis.

[CR71] Rehder B, Florack M, Korte K-R, Schwanholz J (2021). Organisierte Interessen und Staat. Wer gewinnt und wer verliert in der „Coronakratie“?. Coronakratie. Demokratisches Regieren in Ausnahmezeiten.

[CR70] Rehder B, van Elten K (2019). Legal Tech & Dieselgate. Digitale Rechtsdienstleister als Akteure der strategischen Prozessführung. Zeitschrift für Rechtssoziologie.

[CR69] Rehder Britta von Winter T, Willems U (2009). Interessenvermittlung in Politikfeldern. Vergleichende Befunde der Policy- und Verbändeforschung.

[CR72] Sachverständigenrat zur Begutachtung der gesamtwirtschaftlichen Entwicklung. Corona-Krise gemeinsam bewältigen, Resilienz und Wachstum stärken. Jahresgutachten 2020/21. Sachverständigenrat zur Begutachtung der gesamtwirtschaftlichen Entwicklung. 2020. https://www.sachverstaendigenrat-wirtschaft.de/fileadmin/dateiablage/gutachten/jg202021/JG202021_Kurzfassung.pdf. Zugegriffen: 27. Nov. 2020.

[CR73] Sack D, Liebig S, Matiaske W, Rosenbohm S (2017). Methoden und Daten zur Erforschung spezieller Organisationen: Interessenorganisationen. Handbuch Empirische Organisationsforschung.

[CR75] Sack D (2021). Chambers of commerce in Europe. Self-governance and institutional change.

[CR76] Sack D, Fuchs S, von Winter T, von Blumenthal J (2014). Wirtschaftskammern und Parlamente. Einflussmöglichkeiten, Ressourcendependenz und parteipolitische Koalitionen. Interessengruppen und Parlamente.

[CR74] Sack D, Aanor R, Fuchs S (2021). Vom Lockdown in die Staatsbeteiligung? Wirtschaftspolitik in der Covid-19 Pandemie. Der moderne Staat.

[CR77] Sack D, van Elten K, Fuchs S (2014). Legitimität und Self-Governance. Organisationen, Narrative und Mechanismen bei Wirtschaftskammern.

[CR78] Schiffers M (2019). Lobbyisten am runden Tisch: Einflussmuster in Koordinierungsgremien von Regierungen und Interessengruppen.

[CR79] Schmitter PC, Lehmbruch G, Schmitter PC (1982). Reflections on where the theory of neo-corporatism has gone and where the praxis of neo-corporatism may be going. Patterns of corporatist policy-making.

[CR80] Schmitter PC, Streeck W (1999). The organization of business interests. Studying the associative action of business in advanced industrial societies.

[CR81] Schroeder W, Weßels B (2017). Handbuch Arbeitgeber- und Wirtschaftsverbände in Deutschland.

[CR82] Sebaldt M, Straßner A (2004). Verbände in der Bundesrepublik Deutschland. Eine Einführung.

[CR83] Silvia SJ, Schroeder W, Weßels B (2017). Mitgliederentwicklung und Organisationsstärke der Unternehmerverbände. Handbuch Arbeitgeber- und Wirtschaftsverbände in Deutschland.

[CR84] Silvia SJ, Schroeder W (2007). Why are German employers associations declining? Arguments and evidence. Comparative Political Studies.

[CR85] Süddeutsche.de. 2021. Coronakrise. Altmaier kündigt neue Wirtschaftshilfen an. Ein Härtefallfonds ist für bisher vernachlässigte Unternehmen geplant. Verbände fordern weitere Korrekturen. Von Francesca Polistina, 16. Februar 2021. https://www.sueddeutsche.de/politik/coronakrise-altmaier-kuendigt-neue-wirtschaftshilfen-an-1.5208379. Zugegriffen: 15. Apr. 2021.

[CR86] SZ (2020). Wer am lautesten schreit. Abwrackprämie, Elektroautos, Verkehrswende – und jede Menge Jobs in Deutschland: Vor dem Autogipfel im Kanzleramt bringen sich die größte deutsche Industriebranche und Umweltschützer in Position.

[CR87] SZ (2020). Stromschlag für die Lobby. Bund plant hohe Kaufprämien für E-Autos – Verbrenner sollen nicht gefördert werden.

[CR88] Traxler F (2010). The long-term development of organised business and its implications for corporatism. A cross-national comparison of membership, activities and governing capacities of business interest associations, 1980–2003. European Journal of Political Research.

[CR90] Urban H-J, Lehndorff S (2012). Crisis corporatism and trade union revitalisation in Europe. A triumph of failed ideas. European models of capitalism in the crisis.

[CR95] Vorholt D (2019). Konkurrenzausschluss bei deutschen Wirtschaftsverbänden: Eine populationszentrierte Untersuchung.

[CR94] von Winter T, von Winter T, von Blumenthal J (2014). Dimensionen des Korporatismus. Strukturmuster der Verbändebeteiligung in der Gesundheitspolitik. Interessengruppen und Parlamente.

[CR93] von Winter T, Willems U (2007). Interessenverbände in Deutschland. Lehrbuch.

[CR96] ZEIT Online. 2021. Peter Altmaier hält Öffnungen auch bei höherer Inzidenz für möglich. 1. März 2021. https://www.zeit.de/politik/deutschland/2021-03/corona-lockerungen-peter-altmaier-infektionszahlen-massnahmen-landesregierung. Zugegriffen: 20. Mai 2021.

