# Supplementary material for: Corporatism as usual?—Covid-19, organized business interests, and the state
Source: Z Politikwiss. 2021 Nov 9;32(3):601–26. [Article in German] doi: 10.1007/s41358-021-00296-x (PMC8577175; doi:10.1007/s41358-021-00296-x)
Supplement: Supplementary file 1 [file 41358_2021_296_MOESM1_ESM.docx]

**Onlinematerial 1**

**Methodik und Datenerhebung**

**Inhalte**

[1 Erfassung der Staat-Verbände-Interaktion 1](#_Toc73354330)

[1.1 Übersicht der identifizierten Treffen, Gespräche und Foren von Staat und organisierten Wirtschaftsinteressen 2](#_Toc73354331)

[2 Fallstudien – Sample und Stellungnahmen 9](#_Toc73354332)

[2.1 Übersicht der einbezogenen Wirtschaftsverbände 10](#_Toc73354333)

[2.2 Liste der ausgewerteten Verbandsstellungnahmen 11](#_Toc73354334)

# Erfassung der Staat-Verbände-Interaktion

Die hier vorgenommene Erfassung der Staat-Verbände-Interaktionen basiert auf der Auswertung von Pressemitteilungen ausgewählter Bundesministerien im ersten Jahr der Coronakrise (März 2020 – Februar 2021) sowie von Bundestagsdrucksachen. Die Erhebung wurde im Zeitraum März - April 2021 durchgeführt.

Die Untersuchung fokussiert auf die Interaktionen von *Arbeitgeberverbänden*, *Wirtschaftsverbänden* und *Wirtschaftskammern* (Handwerkskammern, Industrie- und Handelskammern)^[[1]](#footnote-1)^ mit der *Bundesregierung*. Kontakte zwischen organisierten Wirtschaftsinteressen und Parlament wurden nicht berücksichtigt.

Für die Erfassung der Treffen, Gespräche und Foren von Bundesregierung und organisierten Wirtschaftsinteressen wurden erstens die online verfügbaren Pressemitteilungen von *Bundeskanzleramt (BKAmt), Bundesministerium für Arbeit und Soziales (BMAS), Bundesministerium für Bildung und Forschung (BMBF), Bundesministerium für Ernährung und Landwirtschaft (BMEL), Bundesministerium der Finanzen (BMF), Bundesministerium für Gesundheit (BMG) und Bundesministerium für Wirtschaft und Energie (BMWE)* ausgewertet.

Zweitens wurde über das ‚Dokumentations- und Informationssystem für Parlamentsmaterialien‘ (DIP) des Deutschen Bundestages (<https://dip.bundestag.de>) nach einschlägigen Bundestagsdrucksachen, die Informationen zur Staat-Verbände-Interaktion (z.B. in Regierungsantworten auf parlamentarische Anfragen) enthalten, gesucht. Das DIP wurde einzeln und in Kombination mit den Begriffen ‚INTERESSENGRUPPEN‘, ‚VERBÄNDE*‘, ‚VERBAND*‘, ‚CORONA‘, ‚COVID-19‘, ‚TREFFEN‘, ‚KONSULTATION*‘ und ‚BERATUNG*‘ durchsucht.

Ergänzende und validierende Onlinerecherchen fanden auf Verbandswebsites, Nachrichten-, Fach- und Branchenportalen statt. In die Auswertung gingen dokumentierte Telefonate zwischen Ministerien und Wirtschaftsverbänden nicht mit ein, da die Datengrundlage zu selektiv ist – nur für *zwei* Gesetzgebungsverfahren (Erstes u. zweites Corona-Steuerhilfegesetz) liegen entsprechende Informationen vor.

## Übersicht der identifizierten Treffen, Gespräche und Foren von Staat und organisierten Wirtschaftsinteressen

| **Datum** | **Bezeichnung** | **Ress-ort(s)** | **Teilnehmende Wirtschafts-verbände (WVb.)** | **Zahl teiln. WVb.** | **COVI/ CORP^a^** | **Que-llen (s.u.)** |
| --- | --- | --- | --- | --- | --- | --- |
| 09.03.20 | Runder Tisch Tourismus | BMWE | BTW, DEHOGA,  DRV, DTV,  DZT, IHA,  RDA | 7 | COVI | 1,2,3 |
| 13.03.20 | Verbändegespräch Corona Kanzleramt | BKAmt, BMF, BMWE, BMG, BMVI, BMAS,  BMI | BDA, BDB,  BDI, BDL,  BGA, BTW,  BVR, DIHK,  DSGV, GDV  VDR, ZDH | 12 | COVI | 4 |
| 16.03.20 | Branchendialog Luftfahrt zum Corona Virus | BMWE | BDL | 5**^b^** | COVI | 5, 6 |
| 18.03.20 | Treffen BMWE, BMAS und Sozialpartner | BMAS, BMWE | BDA | 1 | CORP | 7 |
| 18.03.20 | Verbändegespräch BMAS zum Thema „Absicherung Solo- Selbständige“ | BMAS, BMWE, BMF, Kultur-staats-ministerin | BFB,  BVLivemusik,  DEHOGA,  DIHK, OWUS,  VGSD, ZDH | 7 | COVI | 4 |
| 07.04.20 | Treffen Altmaier u. VDA-Präsidentin zum Beitrag der Automobilwirtschaft zum Gesundheitsschutz | BMWE | VDA | 1 | COVI | 8 |
| 17.04.20 | Gespräch Wirtschaftsverbände | BMWE | AUMA, BDA,  BDEW, BDI,  BDL, BDLI,  BDWI, BFB,  BGA, BGL,  BITKOM,  BTW, BVDS,  BVMW,  DEHOGA,  DIHK, DRV,  DVSI, FamUnt,  GDV, Gesamtmetall,  HDE, Markenverband,  RDA, T+M,  VCI, VDA,  VDMA, VSM,  ZAW, ZDH,  ZGV, ZIA,  ZVEI | 34 | COVI | 4 |
| 24.04.20 | Verbändegespräch Corona Kanzleramt | BKAmt, BMF, BMWE, BMG, BMVI, BMAS,  BMI | BDA, BDB,  BDI, BGA,  BTW, BVR,  DEHOGA,  DIHK, DSGV,  GDV, HDE, VDR,  ZDH | 13 | COVI | 4 |
| 01.05.20**^c^** | Konferenz Netzwerk Mittelstand | BMWE | BAK, BDB, BDI,  BDWI, behv, VdU,  BfB, BGA, BVDS,  BVKAP, BVR, DEHOGA, DIHK,  DRV, DSGV, FamUnt, Franchisevb., IHA,  VDB, Wirtschaftsjunioren  ZDH, ZGV | 23 | CORP | 4 |
| 05.05.20 | Autogipfel | BKAmt | VDA | 1 | COVI | 9 |
| 14.05.20 | Arbeitsgruppe für konjunkturbelebende Maßnahmen | BMWE, BMF, BMVI, BMU, BMAS, BKAmt | VDA | 1 | COVI | 10, 11 |
| 18.05.20 | Verbändegespräch Corona Kanzleramt | BKAmt, BMF, BMWE, BMG, BMVI, BMAS,  BMI | BDA, BDB, BDI,  BDL, BGA, BTW,  BVR, DEHOGA,  DIHK, DSGV, GDV  HDE, VDR, ZDH | 14 | COVI | 4 |
| 26.05.20 | Allianz für Aus- und Weiterbildung | BMWE, weitere BReg | BDA, BFB, DIHK, ZDH | 4 | CORP | 12 |
| 26.05.20 | Arbeitsgruppe für konjunkturbelebende Maßnahmen | BMWE, BMF, BMVI, BMU, BMAS, BKAmt | VDA | 1 | COVI | 11, 13 |
| 12.06.20 | Gespräch Wirtschaftsverbände | BMWE | AUMA, BDA, BDEW, BDI, BDL,  BDLI, BVDS, BDWI, BfB, BGA,  BGL, BITKOM, BTW, BVMW, DEHOGA, DIHK, DRV, FamUnt, GDV, Gesamtmetall,  HDE, Markenverband, RDA, T+M, VCI,  VDMA, VDA, VDSI,  VSM, ZAW, ZDH, ZGV, ZIA, ZVEI | 34 | COVI | 4 |
| 23.06.20 | Leitungsgremium Plattform Industrie 4.0 (zu Corona) | BMWE, BMBF | BDI | 1 | CORP | 14, 15 |
| 25.06.20**^c^** | Gespräch "MittelstandsAllianz" - Bareiß | BMWE | Mitgliedsverbände der MittelstandsAllianz (BVMW) | 30 | CORP | 16, 17, 18,19 |
| 24.08.20 | BMWE und HDE Gespräche | BMWE | HDE | 1 | COVI | 20 |
| 26.08.20 | Gespräch "MittelstandsAllianz" - Bareiß | BMWE | Mitgliedsverbände der MittelstandsAllianz (BVMW) | 30 | CORP | 18,19 |
| 01.09.20**^c^** | Konferenz Netzwerk Mittelstand | BMWE | BAK, BDB, BDI, BDWI, behv, VdU, BfB, BGA, BVDS, BVKAP, BVR, DEHOGA, DIHK, DRV, DSGV, FamUnt, Franchisevb., IHA, VDB, Wirtschaftsjunioren  ZDH, ZGV | 23 | CORP | 4 |
| 07.09.20 | Treffen Altmaier und Logistikbranche | BMWE | AMÖ, BGL, BIEK, BWVL, DSLV | 5 | COVI | 21, 22 |
| 08.09.20 | Konzertierte Aktion Mobilität (3. Sitzung) | BMWE, weitere BReg | VDA | 1 | CORP | 23 |
| 14.10.20 | Runder Tisch Luftfahrtindustrie | BMWE, Länder | VDLI, BDL | 2 | CORP | 24, 25 |
| 22.10.20 | Gespräch Wirtschaftsverbände | BMWE | **^d^** | 35 | COVI | 26 |
| 10.11.20 | Nutzfahrzeuggipfel | BMVI | BDEW, BGL, BMVL, VDA | 4 | CORP | 27 |
| 17.11.20 | Konzertierte Aktion Mobilität (4. Sitzung) | BMWE, weitere BReg | VDA | 1 | CORP | 23 |
| 15.12.20 | Austausch Altmaier und Präsident Gesamtmetall | BMWE | Gesamtmetall | 1 | CORP | 28 |
| 16.12.20 | Beratung Altmaier und Präsident BDA | BMWE | BDA | 1 | CORP | 29 |
| 16.02.21 | Wirtschaftsgipfel | BMWE | BTW, HDE, DEHOGA u.w.**^d^** | 40 | COVI | 30, 31 |
| **Quelle:** Eigene Zusammenstellung. **Anmerkungen:** a) COVI = ad-hoc Treffen zur Krisenbewältigung, CORP = etablierte Formate korporatistischer Staat-Verbände-Interaktion; b) Vb.-Teilnehmer nicht vollständig bekannt, Schätzung auf Grundlage der vorliegenden Informationen; c) Genaues Datum unbekannt, Quartal o. KW bestätigt; d) Zahl teilnehmender Wirtschaftsverbände bekannt, genaue Teilnehmer nicht bekannt. **Einzelbelege:**  1) DTV – Deutscher Tourismusverband. 2020. Statement zum Runden Tisch Tourismuswirtschaft. 9. März 2020. <https://www.deutschertourismusverband.de/presse/pressemitteilungen/pressemitteilungen/aktuelle-pressemitteilungen/article/statement-zum-runden-tisch-tourismuswirtschaft.html>. Zugegriffen: 24. März 2021.  2) DStGB – Deutscher Städte- und Gemeindebund. 2020. Runder Tisch zu Auswirkungen des Coronavirus auf die Tourismuswirtschaft. 13. März 2020. <https://www.dstgb.de/aktuelles/archiv/archiv-2020/Runder%20Tisch%20zu%20Auswirkungen%20des%20Coronavirus%20auf%20die%20Tourismuswirtschaft/>. Zugegriffen: 22. - 26. März 2021.  3) RDA – Internationaler Bustouristik Verband. 2020. Runder Tisch im Bundeswirtschaftsministerium: RDA fordert schnelle Liquiditätshilfen für Bus- und Gruppentouristik. <https://www.presseportal.de/pm/57840/4541829>. Zugegriffen: 22. - 26. März 2021.  4) Deutscher Bundestag, Drucksache 19/22073. Antwort der Bundesregierung auf die Kleine Anfrage der Abgeordneten Claudia Müller, Erhard Grundl, Anja Hajduk, weiterer Abgeordneter und der Fraktion BÜNDNIS 90/DIE GRÜNEN – Drucksache 19/21467. Entstehung der Hilfen für Soloselbständige in der Corona-Pandemie, 02. September 2020.  5) BMWE – Bundesministerium für Wirtschaft und Energie. 2020. Presseöffentliche Termine.  Zeitraum: 14. März 2020 bis 20. März. 2020https://www.bmwi.de/Redaktion/DE/PresseoeffentlicheTermine/presseoeffentliche-termine-2020-1403-2003.html. Zugegriffen: 22. – 26. März 2021.  6) TGL – Technik Gewerkschaft Luftfahrt. 2020. Branchendialog Luftfahrt - Die gemeinsame Position der Gewerkschaften und Arbeitgeber ist klar, die systemrelevante Luftfahrtindustrie muss gestützt werden! 19. März 2020. <https://secure.tgl-online.de/typo/index.php?id=37&tx_news_pi1%5Bnews%5D=105&tx_news_pi1%5Bcontroller%5D=News&tx_news_pi1%5Baction%5D=detail&cHash=2a486ba988365c90a9a37882fb8f2260>. Zugegriffen: 22. - 26. März 2021.  7) BMWE – Bundesministerium für Wirtschaft und Energie. 2020. Treffen mit Sozialpartnern – Auswirkungen der Corona-Epidemie auf Unternehmen und Beschäftigte. Pressemitteilung v. 18. März 2020. <https://www.bmwi.de/Redaktion/DE/Pressemitteilungen/2020/20200318-treffen-mit-sozialpartnern.html>. Zugegriffen: 22. - 26. März 2020.  8) BMWE – Bundesministerium für Wirtschaft und Energie, VDA – Verband der Automobilindustrie. 2020. Minister Altmaier und VDA-Präsidentin Müller würdigen Einsatz der Automobilindustrie in Corona-Krise. Gemeinsame Pressemitteilung v. 7. April 2020. <https://www.bmwi.de/Redaktion/DE/Pressemitteilungen/2020/20200407altmaier-und-vda-praesidentin-mueller-wuerdigen-einsatz-der-automobilindustrie-in-corona-krise.html>. Zugegriffen am: 28. April 2021.  9) BMWE – Bundesministerium für Wirtschaft und Energie. 2020. Altmaier: „Innovationskraft der Automobilindustrie stärken“. Pressemitteilung v. 26. Mai 2020. <https://www.bmwi.de/Redaktion/DE/Pressemitteilungen/2020/20200526-altmaier-innovationskraft-der-automobilindustrie-staerken.html>. Zugegriffen: 25. März 2021.  10) BMWE – Bundesministerium für Wirtschaft und Energie. 2020. Altmaier: „Wir wollen, dass der Wohlstand wieder wächst“. Pressemitteilung v. 14. Mai 2020. <https://www.bmwi.de/Redaktion/DE/Pressemitteilungen/2020/20200514-altmaier-wir-wollen-dass-der-wohlstand-wieder-waechst.html>. Zugegriffen: 25. März 2021.  11) Deutscher Bundestag, Drucksache 19/22363. Antwort der Bundesregierung auf die Kleine Anfrage der Abgeordneten Jan Korte, Dr. Petra Sitte, Friedrich Straetmanns, weiterer Abgeordneter und der Fraktion DIE LINKE – Drucksache 19/21601. Einflussnahme von Interessenvertreterinnen und Interessenvertretern auf den Gesetzentwurf der Bundesregierung – Entwurf eines Zweiten Gesetzes zur Umsetzung steuerlicher Hilfsmaßnahmen zur Bewältigung der Corona-Krise (Zweites Corona-Steuerhilfegesetz) (Bundestagsdrucksache 19/20058), 14. September 2020.  12) BMWE – Bundesministerium für Wirtschaft und Energie. 2020. Duale Ausbildung in der Corona-Krise verlässlich fortführen. Pressemitteilung v. 26. Mai 2020. <https://www.bmwi.de/Redaktion/DE/Pressemitteilungen/2020/20200526-duale-ausbildung-in-der-coronakrise-verlaesslich-ausfuehren.html>. Zugegriffen: 26. März 2021.  13) BMWE – Bundesministerium für Wirtschaft und Energie. 2020. Altmaier: „Innovationskraft der Automobilindustrie stärken“. Pressemitteilung v. 26. Mai 2020. <https://www.bmwi.de/Redaktion/DE/Pressemitteilungen/2020/20200526-altmaier-innovationskraft-der-automobilindustrie-staerken.html>. Zugegriffen: 25. März 2021.  14) BMWE – Bundesministerium für Wirtschaft und Energie, BMF – Bundesministerium für Bildung und Forschung. 2020. Industrie 4.0 kann zur Bewältigung der Krise beitragen. Gemeinsame Pressemitteilung v. 23. Juni 2020. https://www.bmwi.de/Redaktion/DE/Pressemitteilungen/2020/20200623-industrie-4-0-kann-zur-bewaeltigung-der-krise-beitragen.html. Zugegriffen: 26. März 2021.  15) Plattform Industrie 4.0. 2020. Zusammensetzung der Plattform Industrie 4.0. Stand: 22.06.2020. <https://www.plattform-i40.de/PI40/Redaktion/DE/Downloads/Publikation-gesamt/zusammensetzung_plattform.pdf?__blob=publicationFile&v=10>. Zugegriffen: 26. März 2021.  16) VGSD – Verband der Gründer und Selbstständigen Deutschland. 2020. Videokonferenz mit Wirtschafts-Staatssekretär und Mittelstandsbeauftragtem Thomas Bareiß. 26. Juni 2020. <https://www.vgsd.de/videokonferenz-mit-wirtschafts-staatssekretaer-und-mittelstandsbeauftragtem-thomas-bareiss/>. Zugegriffen: 26. März 2021.  17) Deutscher Bundestag, Drucksache 19/25220. Einflussnahme von Interessenvertreterinnen und Interessenvertretern auf den Gesetzentwurf der Bundesregierung – Entwurf eines Gesetzes zur Änderung des Gesetzes gegen Wettbewerbsbeschränkungen für ein fokussiertes, proaktives und digitales Wettbewerbsrecht 4.0 und anderer wettbewerbsrechtlicher Bestimmungen (GWB-Digitalisierungsgesetz) (Bundesratsdrucksache 568/20), 14. Dezember 2020.  18) KKC – Krankenhaus – Kommunikations – Centrum e.V..2020. Digitaler Gesprächstermin mit Thomas Bareiß, Beauftragter der Bundesregierung für den Mittelstand. 26. August 2020. https://www.kkc.info/allgemein/digitaler-gespraechstermin-mit-thomas-bareiss-beauftragter-der-bundesregierung-fuer-den-mittelstand. Zugegriffen: 26. März 2021.  19) bdo – Bundesverband Deutscher Omnibusunternehmer. 2020. Weekly Update. Ausgabe 26/2020. <https://bdo.org/uploads/assets/5ef98dcb8f5d0b7a5a00027b/original/bdo_Weekly_Update_26-2020.pdf?1593413067>. Zugegriffen: 26. März 2021.  20) BMWE – Bundesministerium für Wirtschaft und Energie. 2020. Mittelstandsbeauftragter Bareiß: „Stehe im engen Dialog mit HDE, um Einzelhandel fit für die Zukunft zu machen“. Pressemitteilung v. 24. August 2020. <https://www.bmwi.de/Redaktion/DE/Pressemitteilungen/2020/20200824-bareiss-stehe-im-engen-dialog-mit-hde-um-einzelhandel-fit-fuer-die-zukunft-zu-machen.html>. Zugegriffen: 26. März 2021.  21) VerkehrsRundschau. 2020. Altmaier lobt Logistikbranche. 8. September 2020. <https://www.verkehrsrundschau.de/nachrichten/altmaier-lobt-logistikbranche-2660045.html>. Zugegriffen: 26. März 2021.  22) BMWE – Bundesministerium für Wirtschaft und Energie. 2020. Bundesminister Altmaier tauscht sich mit Vertretern der Logistikverbände über aktuelle Entwicklungen in der Logistikbranche aus. Pressemitteilung v. 7. September 2020. <https://www.bmwi.de/Redaktion/DE/Pressemitteilungen/2020/09/20200907-altmaier-tauscht-sich-mit-vertretern-der-logistikverbaende-aus.html>. Zugegriffen: 26. März 2021.  23) Deutscher Bundestag, Drucksache 19/25739. Antwort der Bundesregierung auf die Kleine Anfrage der Abgeordneten Oliver Luksic, Frank Sitta, Bernd Reuther, weiterer Abgeordneter und der Fraktion der FDP – Drucksache 19/25329. Maßnahmen und Ergebnisse der bisherigen „Autogipfel. 08. Januar 2021.  24) BMWE – Bundesministerium für Wirtschaft und Energie. 2020. Zukunftsperspektiven in der Krise – Runder Tisch Luftfahrtindustrie bekräftigt den Willen, mit Technologie made in Germany die emissionsfreie Luftfahrt der Zukunft zu gestalten. Pressemitteilung v. 14. Oktober 2020. <https://www.bmwi.de/Redaktion/DE/Pressemitteilungen/2020/10/20201014-zukunftsperspektiven-in-der-krise.html>. Zugegriffen: 26. März 2021.  25) BMWE – Bundesministerium für Wirtschaft und Energie. 2020. Kickoff für die Luftfahrt der Zukunft – Runder Tisch Luftfahrtindustrie tagt in Berlin. Pressemitteilung v. 8. Februar 2019. <https://www.bmwi.de/Redaktion/DE/Pressemitteilungen/2019/20190208-kickoff-fuer-die-luftfahrt-der-zukunft.html>. Zugegriffen: 22. - 24. März 2021.  26) BMWE – Bundesministerium für Wirtschaft und Energie. 2020. Bundeswirtschaftsminister Altmaier: „Wirtschaft braucht weiterhin unsere Unterstützung“. Pressemitteilung v. 22. Oktober 2020. <https://www.bmwi.de/Redaktion/DE/Pressemitteilungen/2020/10/20201022-altmaier-wirtschaft-braucht-weiterhin-unsere-unterstuetzung.html>. Zugegriffen: 22. März 2021.  27) BMVI – Bundesministerium für Verkehr und digitale Infrastruktur. 2020b. Nutzfahrzeuggipfel: Mit alternativen Antrieben auf dem Weg zur Nullemissionslogistik auf der Straße. 10. November 2020. <https://www.bmvi.de/SharedDocs/DE/Video/Youtube/nutzfahrzeuggipfel-11-11-2020.html>. Zugegriffen: 28. April 2021.  28) BMWE – Bundesministerium für Wirtschaft und Energie. 2020. Bundeswirtschaftsminister Altmaier tauscht sich mit dem neuen Präsidenten des Arbeitgeberverbandes Gesamtmetall Dr. Wolf aus. Pressemitteilung v. 15. Dezember 2020. <https://www.bmwi.de/Redaktion/DE/Pressemitteilungen/2020/12/20201215-bundeswirtschaftsminister-altmaier-tauscht-sich-mit-dem-neuen-praesidenten-des-arbeitgeberverbandes-gesamtmetall-dr-wolf-aus.html>. Zugegriffen: 22. - 26. März 2021.  29) BMWE – Bundesministerium für Wirtschaft und Energie. 2020. Altmaier und der neue BDA-Präsident Dr. Dulger vereinbaren enge Abstimmung für wirtschaftlichen Aufschwung nach Corona- Pandemie. Pressemitteilung v. 16. Dezember 2020. <https://www.bmwi.de/Redaktion/DE/Pressemitteilungen/2020/12/20201216-altmaier-bda-praesident-dr-dulger-vereinbaren-abstimmung-wirtschaftlichen-aufschwung-corona-pandemie.html>. Zugegriffen: 22. März 2021.  30) BMWE – Bundesministerium für Wirtschaft und Energie. 2021. Presseöffentliche Termine. Zeitraum: 13. Februar 2021 bis 19. Februar. 2021. <https://www.bmwi.de/Redaktion/DE/PresseoeffentlicheTermine/presseoeffentliche-termine-2021-1302-1902.html>. Zugegriffen: 22. - 26. März 2021.  31) BMWE – Bundesministerium für Wirtschaft und Energie. 2021. Altmaier im Vorfeld des heutigen Wirtschaftsgipfels. Pressemitteilung. 16. Februar 2021. https://www.bmwi.de/Redaktion/DE/Pressemitteilungen/2021/02/20210216-altmaier-im-vorfeld-des-heutigen-wirtschaftsgipfels.html. Zugegriffen am: 28. April 2021. | | | | | | |

# Fallstudien – Sample und Stellungnahmen

Die Fallstudienergebnisse basieren auf der qualitativen Inhaltsanalyse online verfügbarer Stellungnahmen von Verbänden und deren Validierung durch die Auswertung von Medienberichten (F.A.Z., SZ).^[[2]](#footnote-2)^ Es wurden die manifesten Inhalte der Stellungnahmen ausgewertet, zusammengefasst und verglichen (Miles et al. 2014)^[[3]](#footnote-3)^. Einbezogen wurden alle Stellungnahmen mit Bezug auf die jeweiligen Phasen der Staat-Verbände-Interaktion von Branchen- und Spitzenverbänden^[[4]](#footnote-4)^ der deutschen Wirtschaft (n = 23).

Das hier berücksichtigte Sample (s. a)) umfasst *erstens* Branchen- und Spitzenverbände der deutschen Wirtschaft, die seit März 2020 regelmäßig an den ‚Verbändegesprächen‘ im Bundeskanzleramt teilnahmen („Spitzentreffen BKanzA“; lt. BT-Drs. 19/22073)^[[5]](#footnote-5)^. *Zweitens* werden, nicht zuletzt aufgrund der ökonomischen Bedeutung des Mittelstands, große Mittelstandsverbände einbezogen, die entweder Teilnehmer des regelmäßig einberufenen „Netzwerk Mittelstand“ im BMWE sind (lt. BT-Drs. 19/22073) oder lt. der Aufzählung in Krickhahn (2017)^[[6]](#footnote-6)^ zusätzlich aufgrund ihrer Größe (Mitgliederzahl) relevant sind. Einbezogen wurden nur „Allgemeine Mittelstandsverbände“ (Krickhahn 2017), d.h. keine branchenspezifischen (z.B. mittelständische Bauunternehmen) oder parteinahen (z.B. Mittelstands- und Wirtschaftsvereinigung der CDU/CSU) Zusammenschlüsse. Ergänzend und *drittens* werden mit dem *Verband der Automobilindustrie (VDA)* und dem *Verband Deutscher Maschinen und Anlagenbau (VDMA)* zwei Verbände berücksichtigt, die in der Coronakrise besonders deutlich und häufig Positionen artikulierten und zugleich große Wirtschaftsbranchen vertreten.

Die Verbandsstellungnahmen (n = 98) wurden durch die eingehende Recherche auf den Websites der Verbände erhoben. Alle Recherchen wurden in den Monaten März - Mai 2021 durchgeführt. Durchsucht wurden jeweils die Startseiten, dezidierte Corona-bezogene Kategorien sowie aktuelle Meldungen, Stellungnahmen und Pressemitteilungen der Verbandswebsites. Einbezogen wurden sodann alle Stellungnahmen in den drei von uns identifizierten Phasen der Staat-Verbände-Interaktion bzw. mit Bezug zu den jeweiligen Schlüsselereignissen: Verabschiedung des Konjunkturpakets (April - Juni 2020) sowie Lockdown-Beschlüsse und Wirtschaftsgipfel (Oktober 2020 - Februar 2021). Für die Automobilindustrie wurden alle Pressemitteilungen des *Verbands der Automobilindustrie (VDA)* zwischen April und Dezember 2020 gesichtet und hinsichtlich dezidierter Forderungen nach politischen Förderprogrammen und Maßnahmen bzw. ihrer Bewertung ausgewählt.

## Übersicht der einbezogenen Wirtschaftsverbände

| **Lfd. Nr.** | **Verband** | **Akronym** | **Begründung Auswahl** |
| --- | --- | --- | --- |
| 1 | Bundesverband der Deutschen Industrie | BDI | Spitzentreffen BKanzA |
| 2 | Bundesverband der Deutschen Tourismuswirtschaft | BTW | Spitzentreffen BKanzA |
| 3 | Bundesverband der Deutschen Volksbanken und Raiffeisenbanken | BVR | Spitzentreffen BKanzA |
| 4 | Bundesverband deutscher Banken | BDB | Spitzentreffen BKanzA |
| 5 | Bundesverband Großhandel, Außenhandel, Dienstleistungen | BGA | Spitzentreffen BKanzA |
| 6 | Bundesvereinigung der Deutschen Arbeitgeberverbände | BDA | Spitzentreffen BKanzA |
| 7 | Deutsche Industrie- und Handelskammertag | DIHK | Spitzentreffen BKanzA |
| 8 | Deutscher Hotel- und Gaststättenverband | DEHOGA | Spitzentreffen BKanzA |
| 9 | Deutscher Sparkassen- und Giroverband | DSGV | Spitzentreffen BKanzA |
| 10 | Gesamtverband der Deutschen Versicherungswirtschaft | GDV | Spitzentreffen BKanzA |
| 11 | Handelsverband Deutschland | HDE | Spitzentreffen BKanzA |
| 12 | Verband Deutscher Reeder | VDR | Spitzentreffen BKanzA |
| 13 | Zentralverband des Deutschen Handwerks | ZDH | Spitzentreffen BKanzA |
| 14 | Deutscher Reiseverband | DRV | Netzwerk Mittelstand |
| 15 | Bundesverband E-Commerce und Versandhandel Deutschland | BEHV | Netzwerk Mittelstand |
| 16 | Bundesverband Deutsche Startups | BVDS | Netzwerk Mittelstand |
| 17 | Die Familienunternehmer | FamUnt | Netzwerk Mittelstand |
| 18 | Der Mittelstandsverbund | ZGV | Netzwerk Mittelstand |
| 19 | Bundesverband der Dienstleistungswirtschaft | BDWI | Netzwerk Mittelstand |
| 20 | Bundesverband mittelständische Wirtschaft | BVMW | Ergänzung nach Krickhahn 2017 |
| 21 | Bundesverband der Selbstständigen | BDS/DGV | Ergänzung nach Krickhahn 2017 |
| 22 | Verband der Automobilindustrie | VDA | Herausgehobene Stellung in öffentlicher Diskussion |
| 23 | Verband Deutscher Maschinen- und Anlagenbau | VDMA | Kritik ab Frühjahr 2020, größter Branchenverband der Industrie |
| **Quelle:** Eigene Zusammenstellung. **Anmerkungen:** „Spitzentreffen BKanzA“ bezeichnet Verbände, die an regelmäßigen Treffen mit Bundeskanzlerin und -ministern im Frühjahr 2020 teilnahmen (lt. BT-Drs. 19/22073); „Netzwerk Mittelstand“ bezeichnet Verbände, die Teilnehmer des regelmäßig einberufenen „Netzwerk Mittelstand“ im BMWE sind (lt. BT-Drs. 19/22073); Weitere Ergänzungen großer Mittelstandsverbände lt. der Aufzählung in Krickhahn 2017 (berücksichtigt wurden „Allgemeine Mittelstandsverbände“) sowie von VDA und VDMA. | | | |

## Liste der ausgewerteten Verbandsstellungnahmen

AGM – Arbeitsgemeinschaft Mittelstand v. 02.06.2020a. Arbeitsgemeinschaft Mittelstand zum Konjunkturpaket der Bundesregierung. https://www.arbeitsgemeinschaft-mittelstand.de/pressemitteilungen/51-arbeitsgemeinschaft-mittelstand-zum-konjunkturpaket-der-bundesregierung/?tx_news_pi1%5Bcontroller%5D=News&tx_news_pi1%5Baction%5D=detail. Zugegriffen: 12. Mai 2021.

AGM – Arbeitsgemeinschaft Mittelstand v. 02.06.2020b. Wertschöpfung und Beschäftigung mit dem Mittelstand stärken. Eckpunkte der Arbeitsgemeinschaft Mittelstand für ein Konjunkturprogramm im Kontext der Corona–Pandemie. https://www.arbeitsgemeinschaft-mittelstand.de/content/Positionen/AGM_Konjunkturpaket_der_Bundesregierung_Wertscho__pfung_und_Bescha__ftigung_....pdf. Zugegriffen: 26. März 2021.

AGM – Arbeitsgemeinschaft Mittelstand v. 07.06.2020. Arbeitsgemeinschaft Mittelstand: Vor allem mit Tempo kann das Konjunkturpaket Wirkung entfalten. https://www.arbeitsgemeinschaft-mittelstand.de/pressemitteilungen/52-arbeitsgemeinschaft-mittelstand-vor-allem-mit-tempo-kann-das-konjunkturpaket-wirkung-entfalten/?tx_news_pi1%5Bcontroller%5D=News&tx_news_pi1%5Baction%5D=detail. Zugegriffen: 12. Mai 2021.

BDA – Bundesvereinigung der Deutschen Arbeitgeberverbände v. 04.06.2020. Arbeitgeberpräsident Kramer: Konjunkturpaket ist wirtschaftliches Kraftpaket und Zeichen von Zuversicht. <https://www.presseportal.de/pm/6512/4614073>. Zugegriffen: 12. Juni 2020.

BDA – Bundesvereinigung der Deutschen Arbeitgeberverbände v. 08.12.2020. Sollten die Beschränkungen von Covid nachlassen, muss auch die staatliche Fürsorge nachlassen. https://arbeitgeber.de/sollten-die-beschraenkungen-von-covid-nachlassen-muss-auch-die-staatliche-fuersorge-nachlassen/. Zugegriffen: 09. April 2021.

BDA – Bundesvereinigung der Deutschen Arbeitgeberverbände v. 16.02.2021. Arbeitgeberpräsident Dulger: Regelmäßige Gespräche mit Regierung sind richtig, ersetzen aber kein mit den Sozialpartnern abgestimmtes Handeln. https://arbeitgeber.de/arbeitgeberpraesident-dulger-regelmaessige-gespraeche-mit-der-regierung-sind-richtig-ersetzen-aber-kein-mit-den-sozialpartnern-abgestimmtes-handeln/. Zugegriffen: 09. April 2021.

BDA – Bundesvereinigung der Deutschen Arbeitgeberverbände v. 29.10.2020. Lockdown nicht resignierend verschlafen – Jetzt die Strategie fortentwickeln. https://arbeitgeber.de/lockdown-nicht-resignierend-verschlafen/. Zugegriffen: 09. April 2021.

BDB – Bundesverband deutscher Banken v. 25.05.2020. Bitkom und Bankenverband fordern schnelle Corona-Hilfe für Startups. https://www.bitkom.org/Presse/Presseinformation/Bitkom-und-Bankenverband-fordern-schnelle-Corona-Hilfe-fuer-Startups, Zugegriffen am 12. Mai 2021.

BDI - Bundesverband der Deutschen Industrie, BDA – Bundesvereinigung der Deutschen Arbeitgeberverbände v. 10.02.2021. BDA und BDI dringen auf evidenzbasiertes Öffnungsszenario und Planungshorizont. https://bdi.eu/artikel/news/deutschland-steht-vor-entscheidenden-wochen-fuer-evidenzbasiertes-oeffnungsszenario-und-planungshori/. Zugegriffen: 22. April 2021.

BDI – Bundesverband der Deutschen Industrie v. 16.04.2020. Neustart und Erholung. https://bdi.eu/publikation/news/neustart-und-erholung/. Zugegriffen: 22. April 2021.

BDI – Bundesverband der Deutschen Industrie v. 16.02.2021. Den Aufschwung unterstützen. https://bdi.eu/publikation/news/den-aufschwung-unterstuetzen/. Zugegriffen: 22. April 2021.

BDI – Bundesverband der Deutschen Industrie v. 28.05.2020. Eckpunkte für ein modernes und effektives Konjunkturpaket. https://bdi.eu/publikation/news/eckpunkte-fuer-ein-modernes-und-effektives-konjunkturpaket/. Zugegriffen: 12. Mai 2021

BDWi – Bundesverband der Dienstleistungswirtschaft v. 12.06.2020. Konjunkturpaket – Videokonferenz mit Bundesminister Altmaier. https://www.bdwi-online.de/presse/pressemeldungen/pressemeldungen/article/konjunkturpaket-videokonferenz-mit-bundesminister-altmaier/. Zugegriffen: 12. Mai 2021.

BDWi – Bundesverband der Dienstleistungswirtschaft v. 15.12.2020. Lockdown im Januar beenden – jetzt die Voraussetzungen schaffen. https://www.bdwi-online.de/presse/pressemeldungen/pressemeldungen/article/lockdown-im-januar-beenden-jetzt-die-voraussetzungen-schaffen/. Zugegriffen: 9. April 2021.

BDWi – Bundesverband der Dienstleistungswirtschaft v. 16.02.2021. Wirtschaftsgipfel im BMWi – BDWi für eine Beendigung des Lockdowns. https://www.bdwi-online.de/presse/pressemeldungen/pressemeldungen/article/wirtschaftsgipfel-im-bmwi-bdwi-fuer-eine-beendigung-des-lockdowns/, Zugegriffen am 9. April 2021.

BDWi – Bundesverband der Dienstleistungswirtschaft v. 28.10.2020. BDWi lehnt einen erneuten Lockdown ab. https://www.bdwi-online.de/presse/pressemeldungen/pressemeldungen/article/bdwi-lehnt-einen-erneuten-lockdown-ab/. Zugegriffen: 9. April 2021.

BEHV – Bundesverband E-Commerce und Versandhandel Deutschland v. 25.11.2020. behv fordert vorübergehende Aufhebung des Verbots der Sonn- und Feiertagsarbeit für den gesamten Handel. https://www.bevh.org/presse/pressemitteilungen/details/bevh-fordert-voruebergehende-aufhebung-des-verbots-der-sonn-und-feiertagsarbeit-fuer-den-gesamten-han.html. Zugegriffen: 9.April 2021.

BEHV – Bundesverband E-Commerce und Versandhandel Deutschland v. 13.12.2020. Versorgung und Existenzen sichern im Lockdown – bevh fordert bundesweit einheitliche Erlaubnis von kontaktlosem Click & Collect und für Arbeit an Sonn- und Feiertagen im Handel, seiner Logistik sowie bei Lieferdiensten. https://www.bevh.org/presse/pressemitteilungen/seite/3.html. Zugegriffen: 9.April 2021.

BGA – Bundesverband Großhandel, Außenhandel, Dienstleistungen v. 04.06.2020. BGA zum Konjunkturpaket: Weniger wäre (noch) mehr gewesen“ https://www.bga.de/presse/pressemitteilungen/. Zugegriffen: 12. Mai 2021.

BGA – Bundesverband Großhandel, Außenhandel, Dienstleistungen v. 28.10.2020. BGA zum bevorstehenden Corona-Gipfel: Nicht das Kind mit dem Bade ausschütten. https://www.bga.de/presse/pressemitteilungen/. Zugegriffen: 9. April 2021.

BGA – Bundesverband Großhandel, Außenhandel, Dienstleistungen v. 03.11.2020. BGA: Teil-Lockdown hat dramatische Folgen für viele Großhandelsbetriebe. https://www.bga.de/presse/pressemitteilungen/. Zugegriffen: 9. April 2021.

BGA – Bundesverband Großhandel, Außenhandel, Dienstleistungen v. 22.02.2021. Altmaier zwischen allen Stühlen. <https://www.bga.de/presse/pressemitteilungen/>. Zugegriffen: 22. April 2021.

BTW – Bundesverband der Deutschen Tourismuswirtschaft, asr – Allianz selbständiger Reiseunternehmen – Bundesverband, BVCD – Bundesverband der Campingwirtschaft in Deutschland, BVDIU – Bundesverband der Deutschen Incoming-Unternehmen, bdo – Bundesverband Deutscher Omnibusunternehmen, DEHOGA – Deutscher Hotel- und Gaststättenverband, Deutscher Ferienhausverband, DRV – Deutscher Reiseverband, DTV – Deutscher Tourismusverband, EVVC – Europäischer Verband der Veranstaltungs-Centren, forum anders reisen, HSMA Deutschland, RDA – Internationaler Bustouristik Verband, Reisenetz – Deutscher Fachverband für Jugendreisen, VIR – Verband Internet Reisevertrieb v. 04.06.2020. Rettungspaket: Wenig Licht, viel Schatten für Tourismuswirtschaft. http://www.btw.de/presse-publikationen/pressemitteilungen/1013-rettungspaket-wenig-licht-viel-schatten-fuer-tourismuswirtschaft.html. Zugegriffen: 25. März 2021.

BTW – Bundesverband der Deutschen Tourismuswirtschaft v. 16.02.2021. „Statement der Tourismuswirtschaft zum ,Wirtschaftsgipfel´“. <http://www.btw.de/presse-publikationen/pressemitteilungen/1132-statement-der-tourismuswirtschaft-zum-wirtschaftsgipfel/print.html>. Zugegriffen: 22. April 2021.

BTW – Bundesverband der Deutschen Tourismuswirtschaft v. 25.11.2020. Bund-Länder-Gespräche: Tourismuswirtschaft darf nicht zum dauerhaften Spielball der Coronapolitik werden. http://www.btw.de/presse-publikationen/pressemitteilungen/1100-bund-laender-gespraeche-tourismuswirtschaft-darf-nicht-zum-dauerhaften-spielball-der-coronapolitik-werden.html . Zugegriffen: 22. April 2021.

BTW – Bundesverband der Deutschen Tourismuswirtschaft v. 27.05.2020. 1,2 Millionen Arbeitsplätze in der Tourismuswirtschaft sind akut gefährdet: Schnelle unbürokratische Beihilfen notwendig. http://www.btw.de/presse-publikationen/pressemitteilungen/1100-bund-laender-gespraeche-tourismuswirtschaft-darf-nicht-zum-dauerhaften-spielball-der-coronapolitik-werden.html. Zugegriffen: 22. April 2021.

BTW – Bundesverband der Deutschen Tourismuswirtschaft v. 27.10.2020. Lockdown Light: Tourismuswirtschaft darf nicht schon wieder zum Bauernopfer der Pandemiepolitik werden. http://www.btw.de/presse-publikationen/pressemitteilungen/1100-bund-laender-gespraeche-tourismuswirtschaft-darf-nicht-zum-dauerhaften-spielball-der-coronapolitik-werden.html. Zugegriffen: 22. April 2021.

BTW – Bundesverband der Deutschen Tourismuswirtschaft v. 29.10.2020. Lockdown ,Light´ trifft die Tourismuswirtschaft schwer“ http://www.btw.de/presse-publikationen/pressemitteilungen/1086-lockdown-light-trifft-die-tourismuswirtschaft-schwer.html. Zugegriffen: 22. April 2021.

BVDS – Bundesverband Deutsche Startups v. 04.06.2020. Startup-Verband: Konjunkturpaket setzt positive Signale für das Startup-Ökosystem. https://deutschestartups.org/2020/06/04/startup-verband-konjunkturpaket-setzt-positive-signale-an-das-startup-oekosystem/. Zugegriffen: 12. Mai 2021.

BVMW – Bundesverband mittelständische Wirtschaft v. 04.06.2020. Ohoven: Konjunkturpaket setzt zu stark auf Konsum. https://www.bvmw.de/news/6155/ohoven-konjunkturpaket-setzt-zu-stark-auf-konsum/. Zugegriffen: 12. Mai 2021.

BVMW – Bundesverband mittelständische Wirtschaft v. 23.06.2020. Mittelstand mit Konjunkturpaket unzufrieden. https://www.bvmw.de/news/6277/mittelstand-mit-konjunkturpaket-unzufrieden/. Zugegriffen: 12. Mai 2021.

BVMW – Bundesverband mittelständische Wirtschaft v. 25.08.2020. Brandbrief an die deutsche Politik. Bundesverband mittelständische Wirtschaft. https://www.bvmw.de/fileadmin/download/BVMW-Brandbrief-Kein-zweiter-Lockdown.pdf. Zugegriffen: 09. April 2021.

BVMW – Bundesverband mittelständische Wirtschaft v. 16.02.2020. Keine Entscheidungen zu Lasten von Wirtschaft und Gesellschaft. https://www.bvmw.de/fileadmln/01- Prnae_uncLNews/Presaemltteilungen/Dateien/PM_34-20_Coronatreffen.pdf. Zugegriffen: 22. April 2021.

BVMW – Bundesverband mittelständische Wirtschaft v. 17.02.2021. Das nächste Treffen sollte ein Mittelstandsgipfel sein. https://www.bvmw.de/fiteadmln /01-Presse_uncLNews/Pressemitteilungen/Dateien/PM_09-21_Altmalef"- Glpfal.pdf. Zugegriffen: 22. April 2021.

BVMW – Bundesverband mittelständische Wirtschaft v. 28.10.2020. BVMW warnt vor Lockdown: „Todesstoß“ für viele Firmen“. https://www.bvmw.de/bvmw-nordbayern/news/7332/bvmw-warnt-vor-lockdown-todestoss-fuer-viele-firmen/. Zugegriffen: 9. April 2021.

BVMW – Bundesverband mittelständische Wirtschaft v. 29.10.2020. Ohoven: Lockdown-Beschlüsse auf Verfassungsmäßigkeit überprüfen. https://www.bvmw.de/news/7351/ohoven-lockdown-beschluesse-auf-verfassungsmaessigkeit-ueberpruefen/. Zugegriffen: 9. April 2021.

BVR - Bundesverband der Deutschen Volksbanken und Raiffeisenbanken v. 04.06.2020. BVR zum Konjunkturpaket: Startschuss für die wirtschaftliche Erholung. https://www.bvr.de/Presse/Pressemitteilungen/BVR_zum_Konjunkturpaket_Startschuss_fuer_die_wirtschaftliche_Erholung. Zugegriffen: 12. Mai 2021.

BVR - Bundesverband der Deutschen Volksbanken und Raiffeisenbanken v. 29.05.2020. BVR zum Konjunkturpaket: Nachfrage- und Investitionsimpulse zielgenau setzen“ https://www.bvr.de/Presse/Pressemitteilungen/BVR_zum_Konjunkturpaket_Nachfrage_und_Investitionsimpulse_zielgenau_setzen. Zugegriffen: 12. Mai 2021.

DEHOGA – Deutscher Hotel- und Gaststättenverband o. D.. Wirtschaftsgipfel: DEHOGA fordert Perspektiven und eine Öffnungsstrategie. https://www.dehoga-bundesverband.de/presse-news/aktuelles/wirtschaftsgipfel-dehoga-fordert-perspektiven-und-eine-oeffnungsstrategie/. Zugegriffen: 22. April 2021.

DEHOGA – Deutscher Hotel- und Gaststättenverband v. 03.11.2020. Lockdown-Hilfen für das Gastgewerbe: Erwartungen der Betreibe dürfen nicht enttäuscht werden. https://www.dehoga-corona.de/aktuelle-dehoga-meldungen-zum-thema-corona-virus/corona-news/detail/news/lockdown-hilfen-fuer-das-gastgewerbe-erwartungen-der-betriebe-duerfen-nicht-enttaeuscht-werden/?tx_news_pi1%5Bcontroller%5D=News&tx_news_pi1%5Baction%5D=detail&cHash=a3f67889007a6634358bb2de6d3e7383. Zugegriffen: 9. April 2021.

DEHOGA – Deutscher Hotel- und Gaststättenverband v. 04.06.2020. Corona-Konjunkturpaket – DEHOGA: Gute und wichtige Impulse, aber Kritik und offene Fragen zum Programm für Überbrückungshilfen. https://www.dehoga-bundesverband.de/presse-news/aktuelles/corona-konjukturpaket-dehoga-gute-und-wichtige-impulse-aber-kritik-und-offene-fragen-zum-programm-fuer-ueberbrueckungshilfen/?fbclid=IwAR3nrFdGeVsK-l8adpdLgGnYp0IeqRcQviSarEXDVSULXVAcKML2tAVSktI. Zugegriffen: 12. Mai 2021.

DEHOGA – Deutscher Hotel- und Gaststättenverband v. 05.11.2020. Lockdown-Entschädigung für das Gastgewerbe – DEHOGA begrüßt Regelungen zur Novemberhilfe. https://www.dehoga-bundesverband.de/index.php?id=2011. Zugegriffen: 9. April 2021.

DEHOGA – Deutscher Hotel- und Gaststättenverband v. 20.05.2020. Dramatischer Umsatzeinbruch im Gastgewerbe – Rettungsfonds ist überfällig. https://www.dehoga-bundesverband.de/presse-news/aktuelles/dramatischer-umsatzeinbruch-im-gastgewerbe-rettungsfonds-ist-ueberfaellig/. Zugegriffen: 12. Mai 2021.

DEHOGA – Deutscher Hotel- und Gaststättenverband v. 27.10.2020. Vor Bund-Länder-Beratungen – Gastgewerbe droht Kollaps. https://www.dehoga-bundesverband.de/presse-news/aktuelles/vor-bund-laender-beratungen-gastgewerbe-droht-kollaps/. Zugegriffen: 9. April 2021.

DIHK – Deutsche Industrie- und Handelskammertag, o. D.. Wirtschaftsstandort bewahren – Zukunftschancen ergreifen. https://www.dihk.de/de/aktuelles-und-presse/coronavirus/5-punkte. Zugegriffen: 12. Mai 2021.

DRV – Deutscher Reiseverband v. 02.06.2020. Verbraucherschützer und Reisewirtschaft fordern Fonds zur Absicherung der Kundengeldrückerstattung. https://www.drv.de/anzeigen/txnews/verbraucherschuetzer-und-reisewirtschaft-fordern-fonds-zur-absicherung-der-kundengeldrueckerstattung.html. Zugegriffen: 12. Mai 2021.

DRV – Deutscher Reiseverband v. 05.06.2020. Maßnahmen aus Konjunkturpaket für Ausbildungsbetriebe reichen nicht aus. https://www.drv.de/anzeigen/txnews/massnahmen-aus-konjunkturpaket-fuer-ausbildungsbetriebe-reichen-nicht-aus.html. Zugegriffen: 12. Mai 2021.

DRV – Deutscher Reiseverband v. 16.02.2021. Wirtschaftsgipfel: Deutscher Reiseverband fordert Restart-Strategie. https://www.drv.de/anzeigen/txnews/wirtschaftsgipfel-deutscher-reiseverband-fordert-restart-strategie.html. Zugegriffen: 22. April 2021.

DSLV – Bundesverband Spedition und Logistik v. 11.11.2020. DSLV zufrieden mit BMVI-Nutzfahrzeuggipfel: Förderkulisse aufbauen, Marktrisiken abfedern, Planungssicherheit herstellen. https://www.dslv.org/dslv/web.nsf/id/li_fdihbv9kru.html. Zugegriffen: 25. März 2021.

FamUnt – Die Familienunternehmer v. 04.06.2020. Familienunternehmer von Konjunkturpaket positiv und negativ überrascht. https://www.familienunternehmer.eu/regionalkreis-dortmund/presse/pressemitteilungen/detail/article/familienunternehmer-von-konjunkturpaket-positiv-und-negativ-ueberrascht.html. Zugegriffen: 12. Mai 2021.

FamUnt – Die Familienunternehmer v. 08.02.2021. Familienunternehmer fordern Corona-Wirtschaftsgipfel. https://www.familienunternehmer.eu/presse-news/pressemitteilungen/detail/article/familienunternehmer-fordern-corona-wirtschaftsgipfel.html. Zugegriffen: 22. April 2021.

FamUnt – Die Familienunternehmer v. 14.12.2020. Familienunternehmer zu hartem Lockdown: viele Fragen, wenig Antworten. https://www.familienunternehmer.eu/regionalkreis-hamm-ruhr-lippe/presse/pressemitteilungen/detail/article/familienunternehmer-zum-harten-lockdown-viele-fragen-wenig-antworten.html. Zugegriffen: 22. April 2021.

FamUnt – Die Familienunternehmer v. 16.02.2021. Familienunternehmer zum Corona-Wirtschaftsgipfel: Hausaufgaben nicht gemacht“. https://www.familienunternehmer.eu/presse-news/pressemitteilungen/detail/article/familienunternehmer-zum-corona-wirtschaftsgipfel-hausaufgaben-nicht-gemacht.html. Zugegriffen: 22. April 2021.

BVMW – Bundesverband mittelständische Wirtschaft v. 24.11.2021. Mittelstand mahnt dringende Nachbesserungen bei ,Novemberhilfen´. https://www.bvmw.de/news/7592/mittelstand-mahnt-dringende-nachbesserungen-bei-novemberhilfen-an/. Zugegriffen: 22. April 2021.

HDE – Handelsverband Deutschland, o. D.. Beschluss des Koalitionsausschusses vom 3. Juni 2020 zu einem Konjunktur- und Krisenbewältigungspaket – HDE-Bewertung der handelsrelevanten Maßnahmen. <https://www.vds-sportfachhandel.de/wp-content/uploads/2020/06/Themensammlung-Coronavirus_HDE-Forderungen-an-die-Politik.pdf>. Zugegriffen: 31. März 2021.

HDE – Handelsverband Deutschland v. 04.06.2020. Konjunkturpaket setzt positive Impulse für den Handel. https://einzelhandel.de/presse/aktuellemeldungen/12745-konjunkturpaket-setzt-positive-impulse- fuer-den-handel. Zugegriffen: 12. Mai 2021.

HDE – Handelsverband Deutschland v. 05.11.2020. Teil-Lockdown verdirbt Innenstadthandel das Weihnachtsgeschäft. https://einzelhandel.de/presse/pressearchiv/1410-pressemitteilungen-2020/13012-hde-prognose-1-2-prozent-fuer-november-und-dezember. Zugegriffen: 22. April 2021.

HDE – Handelsverband Deutschland v. 08.12.2020. Corona-Eindämmung: Handel wart vor Lockdown mit Ladenschließungen. https://einzelhandel.de/presse/pressearchiv/1410-pressemitteilungen-2020/13072-corona-eindaemmung-handel-warnt-vor-lockdown-mit-ladenschliessungen. Zugegriffen: 22. April 2021.

HDE - Handelsverband Deutschland v. 11.12.2020. Umsatzausfälle nicht mehr aus eigener Kraft zu stemmen: Handel fordert im Falle eines Lockdowns mit Geschäftsschließungen staatliche Hilfen. https://einzelhandel.de/presse/pressearchiv/1410-pressemitteilungen-2020/13075-umsatzausfaelle-nicht-mehr-aus-eigener-kraft-zu-stemmen-handel-fordert-im-falle-eines-lockdowns-mit-geschaeftsschliessungen-staatliche-hilfen. Zugegriffen: 22. April 2021.

HDE – Handelsverband Deutschland v. 13.12.2020. Lockdown im Nicht-Lebensmittelhandel: Mehr Hilfsgelder für Händler und Innenstädte gefordert. https://einzelhandel.de/presse/pressearchiv/1410-pressemitteilungen-2020/13077-lockdown-im-nicht-lebensmittelhandel-mehr-hilfsgelder-fuer-haendler-und-innenstaedte-gefordert. Zugegriffen: 22. April 2021.

HDE – Handelsverband Deutschland v. 14.05.2020. Corona-Konjunkturpaket: Einzelhandel und Verbraucherschützer fordern Entlastung bei Strompreisen. https://einzelhandel.de/presse/aktuellemeldungen/12710-corona-konjunkturpaket-einzelhandel- und-verbraucherschuetzer-fordern-entlastung-bei-strompreisen. Zugegriffen: 12. Mai 2021.

HDE – Handelsverband Deutschland v. 15.02.2021. Vor morgigen Wirtschaftsgipfel: Handel fordert transparenten Stufenplan zur Wiedereröffnung und wirksame Corona-Hilfen. https://einzelhandel.de/presse/aktuellemeldungen/13182-vor-dem-morgigen-wirtschaftsgipfel-handel-fordert-transparenten-stufenplan-zur-wiedereroeffnung-und-wirksame-corona-hilfen. Zugegriffen: 22. März 2021.

HDE – Handelsverband Deutschland v. 16.02.2021. Nach dem Wirtschaftsgipfel: HDE begrüßt Hilfszusagen für größere Handelsunternehmen - Öffnungsperspektive fehlt weiterhin. https://einzelhandel.de/presse/aktuellemeldungen/13183-nach-dem-wirtschaftsgipfel-hde-begruesst-hilfszusagen-fuer-groessere-handels-unternehmen-oeffnungsperspektive-fehlt-weiterhin. Zugegriffen: 22. März 2021.

HDE – Handelsverband Deutschland v. 26.05.2020. Konjunkturprogramm für Handel und Binnenwirtschaft. <https://einzelhandel.de/konjunkturprogramm>. Zugegriffen: 31. März 2021.

HDE – Handelsverband Deutschland v. 26.11.2020. Verlängerung des Lockdown-light verschärft Existensnot bei Händlern in den Innenstädten – Weitere Umsatzverschiebungen in den Online-Handel. https://einzelhandel.de/presse/pressearchiv/1410-pressemitteilungen-2020/13048-verlaengerung-des-lockdown-light-verschaerft-existenznot-bei-haendlern-in-den-innenstaedten-weitere-umsatzverschiebungen-in-den-online-handel. Zugegriffen: 22. April 2021.

HDE – Handelsverband Deutschland v. 27.04.2020. Coronaschecks: Konjunkturpaket für Handel und Binnenwirtschaft. <https://einzelhandel.de/>coronaschecks. Zugegriffen: 31. März 2021.

HDE – Handelsverband Deutschland v. 27.05.2020. HDE fordert Konjunkturprogramm mit Coronaschecks und Überbrückungshilfen. https://einzelhandel.de/presse/aktuellemeldungen/12719-hde-fordert-konjunkturprogramm-mit- coronaschecks-und-ueberbrueckungshilfen. Zugegriffen: 12. Mai 2021.

HDE – Handelsverband Deutschland v. 28.10.2020. Neue Maßnahmen zur Eindämmung der Pandemie – faktischer Lockdown für den Einzelhandel – Viele Händler auf staatliche Hilfe angewiesen. https://einzelhandel.de/presse/pressearchiv/1410-pressemitteilungen-2020/12991-neue-massnahmen-zur-eindaemmung-der-pandemie-faktischer-lockdown-fuer-den-einzelhandel-viele-haendler-auf-staatliche-hilfen-angewiesen. Zugegriffen: 22. April 2021.

HDE – Handelsverband Deutschland v. 28.10.2020. Vor Corona-Gipfel: Einkaufen in der Pandemie sicher – kein Anlass für neuerliche Einschränkungen. https://einzelhandel.de/presse/pressearchiv/1410-pressemitteilungen-2020/12985-vor-corona-gipfel-einkaufen-in-der-pandemie-sicher-kein-anlass-fuer-neuerliche-einschraenkungen. Zugegriffen: 22. April 2021.

VDA – Verband der Automobilindustrie v. 02.09.2020. VDA-Geschäftsführer Dr. Koers: Erneuter Einbruch bei Produktion und Nachfrage muss mit allen Mitteln verhindert werden. https://www.vda.de/de/presse/Pressemeldungen/200902-VDA-Gesch-ftsf-hrer-Dr-Koers-Erneuter-Einbruch-bei-Produktion-und-Nachfrage-muss-mit-allen-Mitteln-verhindert-werden.html. Zugegriffen: 25. März 2021

VDA – Verband der Automobilindustrie v. 04.06.2020. Statement von VDA-Präsidentin Hildegard Müller zum Ergebnis des Koalitionsausschusses. https://www.vda.de/de/presse/Pressemeldungen/200604-Statement-von-VDA-Praesidentin-Hildegard-Mueller-zum-Koalitionsausschuss.html. Zugegriffen: 25. März 2021.

VDA – Verband der Automobilindustrie v. 06.10.2020. Hildegard Müller: Förderung des privaten Ladens setzt an der richtigen Stelle an. https://www.vda.de/de/presse/Pressemeldungen/201006-Hildegard-M-ller-F-rderung-des-privaten-Ladens-setzt-an-der-richtigen-Stelle-an.html. Zugegriffen: 25. März 2021.

VDA – Verband der Automobilindustrie v. 07.04.2020. Minister Altmaier und VDA-Präsidentin Müller würdigen Einsatz der Automobilindustrie in Corona-Krise. https://www.vda.de/de/presse/Pressemeldungen/200407-Minister-Altmaier-und-VDA-Praesidentin-M-ller-w-rdigen-Einsatz-der-Automobilindustrie-in-Corona-Krise.html. Zugegriffen: 25. März 2021.

VDA – Verband der Automobilindustrie v. 08.07.2020. Ausbau der privaten Ladeinfrastruktur ist zentraler Hebel für den Hochlauf der Elektromobilität“ [gemeinsame Stellungnahme mit dem Bundesverband der Energie- und Wasserwirtschaft (BDEW) und dem Bundesverband deutscher Wohnungs- und Immobilienunternehmen (GdW)], https://www.vda.de/de/presse/Pressemeldungen/200708-Ausbau-der-privaten-Ladeinfrastruktur-ist-zentraler-Hebel-f-r-den-Hochlauf-der-Elektromobilit-t.html. Zugegriffen: 25. März 2021.

VDA – Verband der Automobilindustrie v. 08.07.2020. Gemeinsame Empfehlung zum Laden in Wohnanlagen. https://www.vda.de/de/services/Publikationen/gemeinsame-empfehlungen-von-vda%2C-bdew-und-gdw-zum-laden-in-wohnanlagen.html. Zugegriffen: 25. März 2021.

VDA – Verband der Automobilindustrie v. 09.04.2020. Stationären Autoverkauf schnellstmöglich wieder erlauben. https://www.vda.de/de/presse/Pressemeldungen/200409-Gemeinsame-PM-VDA-VDIK-ZDK-und-IG-Metall-Stationaeren-Autoverkauf-schnellstm-glich-wieder-erlauben.html. Zugegriffen: 25. März 2021.

VDA – Verband der Automobilindustrie v. 13.11.2020. E-Ladeinfrastruktur ausbauen – Digitalisierung vorantreiben – Standort Deutschland sichern. https://www.vda.de/de/presse/Pressemeldungen/201113-E-Ladeinfrastruktur-ausbauen---Digitalisierung-vorantreiben---Standort-Deutschland-sichern.html. Zugegriffen: 25. März 2021.

VDA – Verband der Automobilindustrie v. 21.09.2020. Erleichterungen beim Aufbau privater Ladesäulen zügig durch Förderprogramme flankieren. https://www.vda.de/de/presse/Pressemeldungen/200921-Erleichterungen-beim-Aufbau-privater-Lades-ulen-z-gig-durch-F-rderprogramme-flankieren.html. Zugegriffen: 25. März 2021.

VDA – Verband der Automobilindustrie v. 23.10.2020. EU-Klimaziele erfordern raschen Ausbau von E- Ladesäulen-Netz für schwere Lkw. https://www.vda.de/de/presse/Pressemeldungen/201023-EU-Klimaziele-erfordern-raschen-Ausbau-von-E-Lades-ulen-Netz-f-r-schwere-Lkw.html. Zugegriffen: 25. März 2021.

VDA – Verband der Automobilindustrie v. 24.09.2020. Mittelstand muss Fundament der deutschen Wirtschaft bleiben. https://www.vda.de/de/presse/Pressemeldungen/200924-Mittelstand-muss-Fundament-der-deutschen-Industrie-bleiben.html. Zugegriffen: 25. März 2021.

VDA -– Verband der Automobilindustrie v. 28.10.2020. VDA-Position zum 2030 Climate Target Plan“ https://www.vda.de/de/services/Publikationen/vda-position-zum-2030-climate-target-plan.html. Zugegriffen: 25. März 2021.

VDMA – Verband Deutscher Maschinen- und Anlagenbau v. 24.03.2020. Hilfen sind wichtig – aber keine Staatsbeteiligung ohne Ablaufdatum. <https://bayern.vdma.org/viewer/-/v2article/render/47744009>. Zugegriffen: 19. November 2020.

VDMA – Verband Deutscher Maschinen- und Anlagenbau v. 09.12.2020. VDMA: Den Weg aus Corona heraus gestalten!. <http://bildung.vdma.org/viewer/-/v2article/render/57265913>. Zugegriffen: 9. April 2020.

VDMA – Verband Deutscher Maschinen- und Anlagenbau v. 13.12.2020. Maschinenbau unterstützt Corona-Beschlüsse. https://www.vdma.org/viewer/-/v2article/render/4753554. Zugegriffen: 9. April 2020.

VDMA – Verband Deutscher Maschinen- und Anlagenbau v. 29.10.2020. Corona-Maßnahmen weisen den richtigen Weg!. <http://bayern.vdma.org/viewer/-/v2article/render/54649433>. Zugegriffen: 9. April 2020.

ZDH – Zentralverband des Deutschen Handwerks v. 04.06.2020. Krisenbewältigung, Konjunkturstärkung und Zukunftssicherung: zielführender Dreiklang der Koalitionspartner. https://www.zdh.de/presse/pressemitteilungen/krisenbewaeltigung-konjunkturstaerkung-und-zukunftssicherung-zielfuehrender-dreiklang-der-koalitionspartner/?L=0. Zugegriffen: 12. Mai 2021.

ZDH – Zentralverband des Deutschen Handwerks v. 11.11.2020. Teil-Lockdown bremst Erholungsprozess auch im Handwerk deutlich. https://www.zdh.de/presse/pressemitteilungen/teil-lockdown-bremst-erholungsprozess-auch-im-handwerk-deutlich/?L=0. Zugegriffen: 9. April 2021.

ZDH – Zentralverband des Deutschen Handwerks v. 12.06.2020. Konjunkturpaket jetzt schnell und bürokratiearm umsetzen. https://www.zdh.de/presse/pressemitteilungen/konjunkturpaket-jetzt-schnell-und-buerokratiearm-umsetzen/?L=0. Zugegriffen: 12. Mai 2021

ZDH – Zentralverband des Deutschen Handwerks v. 14.05.2020. Konjunkturstärkung auch bei deutlich sinkenden Steuereinnahmen möglich!. https://www.zdh.de/presse/pressemitteilungen/konjunkturstaerkung-auch-bei-deutlich-sinkenden-steuereinnahmen-moeglich/?L=0. Zugegriffen: 12. Mai 2021.

ZDH – Zentralverband des Deutschen Handwerks v. 15.02.2021. Betriebe brauchen konkretisierte Öffnungsperspektiven!. https://www.zdh.de/presse/interviews-statements/betriebe-brauchen-konkretisierte-oeffnungsperspektiven/?L=0. Zugegriffen: 22. April 2021.

ZDH – Zentralverband des Deutschen Handwerks v. 16.02.2021. Betriebe-Schutz ist unabdingbarer Bestandteil der Pandemiebewältigung. https://www.zdh.de/presse/pressemitteilungen/betriebe-schutz-ist-unabdingbarer-bestandteil-der-pandemiebewaeltigung/. Zugegriffen: 22. April 2021.

ZDH – Zentralverband des Deutschen Handwerks v. 22.02.2021. Eine Fachkräftekrise müssen wir unbedingt abwenden. https://www.zdh.de/presse/interviews-statements/eine-fachkraeftekrise-muessen-wir-unbedingt-abwenden/?L=0. Zugegriffen: 22. April 2021.

ZDH – Zentralverband des Deutschen Handwerks v. 25.11.2020. Verlängerungen und Verschärfungen des Teil-Lockdowns trifft viele Betriebe sehr hart. https://www.zdh.de/presse/pressemitteilungen/verlaengerung-und-verschaerfung-des-teil-lockdowns-trifft-viele-betriebe-sehr-hart/?L=0. Zugegriffen: 9. April 2021.

ZDH – Zentralverband des Deutschen Handwerks v. 28.10.2020. Erkennbare Bemühen zur Vermeidung des pauschalen Wirtschafts-Lockdown. https://www.zdh.de/presse/pressemitteilungen/erkennbares-bemuehen-zur-vermeidung-eines-pauschalen-wirtschafts-lockdown/?L=0. Zugegriffen: 9. April 2021.

ZGV – Der Mittelstandsverbund v. 04.06.2020. Konjunkturprogramm mit Mut – aber Lücken für den Mittelstand. https://www.mittelstandsverbund.de/presse/presseinformationen/d-konjunkturprogramm-mit-mut-aber-luecken-fuer-den-mittelstand-1027596695?p=2. Zugegriffen: 12. Mai 2021.

ZGV – Der Mittelstandsverbund v. 09.06.2020. Konjunkturprogramm droht Wettbewerbskraft mittelständischer Fachhändler zu schwächen – Rekapitalisierungsprogramm dringend erforderlich. https://www.mittelstandsverbund.de/presse/presseinformationen/d-konjunkturprogramm-droht-wettbewerbskraft-mittelstaendischer-fachhaendler-zu-schwaechen-rekapitalisierungsprogramm-dringend-erforderlich-1559464645?p=2. Zugegriffen: 12. Mai 2021.

ZGV – Der Mittelstandsverbund v. 13.12.2020. Erneuter Lockdown bringt Mittelstand in existenzielle Bedrängnis – Hilfsinstrumente schnellstens nachjustieren. https://www.mittelstandsverbund.de/presse/presseinformationen/d-erneuter-lockdown-bringt-mittelstand-in-existenzielle-bedraengnis-hilfsinstrumente-schnellstens-nachjustieren-446408812?p=2. Zugegriffen: 9. April 2021.

ZGV – Der Mittelstandsverbund v. 29.05.2020. MITTELSTANDSVERBUND begrüßt neue Überbrückungshilfen – Lücke im KfW-Schnellkredit-Programm sofort schließen. https://www.mittelstandsverbund.de/presse/presseinformationen/d-mittelstandsverbund-begruesst-neue-ueberbrueckungshilfen-luecke-im-kfw-schnellkredit-programm-sofort-schliessen-1636510665?p=3. Zugegriffen: 12. Mai 2021.

1. Wir folgen mit Blick auf die Verbandstypen der in der deutschen Wirtschaftsverbandsforschung etablierten Unterscheidung dreier ‚Säulen‘ organisierter Wirtschaftsinteressen, nämlich von Arbeitgeberverbänden, Wirtschaftsverbänden (jeweils mit freiwilliger Mitgliedschaft) und pflichtmitgliedschaftlich organisierten Wirtschaftskammern. [↑](#footnote-ref-1)
2. Zur Diskussion des Nutzens online verfügbarer Stellungnahmen in der Interessengruppenforschung siehe Rasch, Daniel, Florian Spohr, Rainer Eising, und Simon Ress. 2020. Uncovering interest group participation in Germany: web collection of written statements in ministries and the parliament. *Interest Groups & Advocacy* 9(2020):330-341. https://doi.org/10.1057/s41309-020-00099-5. [↑](#footnote-ref-2)
3. Miles, Matthew B., A. Michael Huberman, und Johnny Saldaña. 2014. *Qualitative Data Analysis. A Methods Sourcebook*. Third Edition. Thousand Oaks, CA: SAGE. [↑](#footnote-ref-3)
4. Für die Unterscheidung der Verbandstypen folgen wir der Terminologie und Systematik bei Kohler-Koch, Beate, Sebastian Fuchs, und David A. Friedrich. 2021. *Verbände mit Zukunft? Die Re-Organisation industrieller Interessen in Deutschland.* Wiesbaden: Springer VS. Im Erscheinen. [↑](#footnote-ref-4)
5. Deutscher Bundestag, Drucksache 19/22073. Antwort der Bundesregierung auf die Kleine Anfrage der Abgeordneten Claudia Müller, Erhard Grundl, Anja Hajduk, weiterer Abgeordneter und der Fraktion BÜNDNIS 90/DIE GRÜNEN – Drucksache 19/21467. Entstehung der Hilfen für Soloselbständige in der Corona-Pandemie, 02. September 2020. [↑](#footnote-ref-5)
6. Krickhahn, Thomas. 2017. Mittelstandsverbände in Deutschland. In *Handbuch Arbeitgeber- und Wirtschaftsverbände in Deutschland*, Hrsg. Wolfgang Schroeder und Bernhard Weßels, 111-140. Wiesbaden: Springer VS. [↑](#footnote-ref-6)
